# Theoretical and Experimental Studies of the Structural Chameleon EuYCuTe_3_

**DOI:** 10.3390/ma18040820

**Published:** 2025-02-13

**Authors:** Anna V. Ruseikina, Maxim V. Grigoriev, Vladimir A. Chernyshev, Evgenii M. Roginskii, Alexander A. Garmonov, Ralf J. C. Locke, Thomas Schleid

**Affiliations:** 1 School of Natural Sciences, University of Tyumen, Tyumen 625003, Russia; maxgirgmvv@ya.ru (M.V.G.); gamma125@mail.ru (A.A.G.); 2 Institute for Inorganic Chemistry, University of Stuttgart, D-70569 Stuttgart, Germany; ralf.locke@iac.uni-stuttgart.de; 3 Institute of Natural Sciences and Mathematics, Ural Federal University Named after the First President of Russia B.N. Yeltsin, Ekaterinburg 620002, Russia; vchern@inbox.ru; 4 Ioffe Institute, Politekhnicheskaya 26, St. Petersburg 194021, Russia; e.roginskii@mail.ioffe.ru

**Keywords:** quaternary tellurides, yttrium, synthesis, crystal structures, magnetic properties, DFT calculations

## Abstract

Layered orthorhombic single crystals of EuYCuTe_3_ are synthesized using the ampoule method from the elemental precursors taken in the ratio of 1 Eu:1 Y:1 Cu:3 Te by heating up to 1120 K with an excess of CsI as flux. The orthorhombic structure of EuYCuTe_3_ is established, and structural parameters are obtained using X-ray diffraction. At ambient conditions, the sample crystallizes in the space group *Pnma* with the unit cell parameters *a* = 11.2730(7) Å, *b* = 4.3214(3) Å, *c* = 14.3271(9) Å. The structure is composed of vertex-connected [CuTe_4_]^7−^ tetrahedra, which form chains along the [010] direction, and of edge-connected [YTe_6_]^9−^ octahedra, which form layers parallel to the (010) plane. The Eu^2+^ cations are found in a capped trigonal prismatic coordination of Te^2−^ anions. The structural phase transition from the *α* to the *β* phase is discovered upon heating the sample to 323 K, which comes accompanied with a decrease of [CuTe_4_]^7−^ tetrahedral distortion. The symmetry of the high-temperature phase is established as ordered in the space group *Cmcm* (*a* = 4.3231(3) Å, *b* = 14.3328(9) Å, *c* = 11.2843(7) Å). The nature and microscopic mechanism of the phase transition is discussed. By cooling it down below 3 K, the soft ferromagnetic properties of EuYCuTe_3_ are discovered. The correlation of the ferromagnetic transition temperature in the series of chalcogenides EuYCu*Ch*_3_ (*Ch* = S, Se, Te) with the ionic radius of the chalcogenide anion is established. The structural dynamical elastic properties of *α*- and *β*-EuYCuTe_3_ were calculated within the ab initio approach. The vibrational mode frequencies and decomposition on irreducible representations, as well as the degree of ion involvement in each mode, were determined. The calculations reveal an imaginary mode in the Y-point of the *Brillouin* zone in the high symmetry *β*-EuYCuTe_3_ phase. This finding explains the nature of structural reconstruction in EuYCuTe_3_ crystal as a second-order phase transition induced by soft mode condensation at the edge of the *Brillouin* zone. The exfoliation of a single layer is simulated theoretically. The exfoliation energy is estimated, and the dynamical properties of EuYCuTe_3_ single layers are studied.

## 1. Introduction

Layered structures attract significant interest due to their great potential for technical applications in submicron electronics as flexible and tunable electronics, photovoltaic and sensor elements [1,2,3]. Copper rare-earth metal chalcogenides are of particular interest because of the high mobility of Cu^+^, which leads to “phonon-liquid electron-crystal” behavior, fast ionic conductivity, low heat capacity, structural disorder, superionicity and promising thermoelectric properties [4,5,6]. In the structures of heterometallic quaternary chalcogenides, anionic two-dimensional layers are formed by the coordination polyhedra of chalcogenide about the rare-earth metals and [Cu*Ch*_4_]^7−^ [7,8,9,10,11,12,13,14,15,16,17,18,19,20,21]. Layered crystalline structures of the yttrium family *M*^2+^Cu^+^Y^3+^(*Ch*^2−^)_3_ (*Ch* = S, Se, Te) exhibit remarkable flexibility regarding the substitution of cations *M*^2+^, leading to the formation of isostructural compounds or a symmetry reduction from the space group *Cmcm* to *Pnma*. A reduction of the radius *r_i_*(*M*^2+^) by more than 12% (*r_i_*(Ba^2+^) = 1.35 Å vs. *r_i_*(Sr^2+^) = 1.18 Å [22]) is accompanied by a change in the symmetry of the quaternary yttrium chalcogenides. Examples include compounds containing alkaline earth metals: BaYCu*Ch*_3_ (*Ch* = S [23,24], Se [4,24], Te [7]) and SrCuY*Ch*_3_ (*Ch* = S [25], Se [4,26]), crystallizing in different space groups: *Cmcm* and *Pnma*, respectively. Closer proximity of the ionic radii *r_i_*(*M*^2+^) (for example, *r_i_*(Sr^2+^) = 1.18 Å vs. *r_i_*(Eu^2+^) = 1.17 Å) [22] facilitates the formation of isostructural compounds, such as SrCuY*Ch*_3_ [4,25,26] and EuYCu*Ch*_3_ (*Ch* = S [8], Se [27]), which crystallize in the same space group (*Pnma*) and structure type. The *M*^2+^ cations in the *M*YCu*Ch*_3_ structures are weakly bound to the crystal lattice as compared to the Cu^+^, Y^3+^ and Ch^2−^ ions, which participate in directed polar covalent bonding [5,6,28]. In the *M*YCu*Ch*_3_ compounds, there is a tightly bound sublattice [YCu*Ch*_3_]^2−^, which can lead to improved electric transport properties, while the weakly bound cations *M*^2+^ help to induce low thermal lattice conductivity [5,6,28,29,30], making these compounds potential thermoelectrics [6].

Solid-state yttrium chalcogenides are an attractive subject, not only because of their interesting structural features but also due to their desirable physical properties, which show a wide potential for applications. For example, EuYCu*Ch*_3_ (*Ch* = S [8], Se [27]) demonstrates a ferromagnetic transition at low temperatures, BaYCuS_3_ exhibits high thermal stability [23], and *M*YCu*Ch*_3_ (*M* = Eu [8,27], Sr [26], Ba [23]; *Ch* = S, Se) displays semiconducting properties, with the compound EuYCuSe_3_ showing band–gap values relevant for photovoltaic materials [27], and BaYCuTe_3_ exhibiting respectable thermoelectric properties [6]. To date, most known quaternary yttrium chalcogenides containing both *d*- and *f*-metals are sulfides and selenides, with significantly fewer tellurides reported. Due to the more diffuse nature of the 5*p* orbitals, lower electronegativity and larger ionic radius of Te^2−^, tellurides often exhibit different structures and properties than sulfides and selenides [19]. The wide range of interactions (about 4 Å), intermediate between the length of a single Te−Te bond and *van-der-Waals* interactions, shown by tellurium affects the structural chemistry and physical properties of tellurides [19]. No information has been found in the literature regarding attempts to synthesize and investigate the telluride EuYCuTe_3_.

In this article, we provide a detailed description of the synthesis, structural characterization and magnetic properties of two polymorphic modifications of EuYCuTe_3_, which have been experimentally obtained for the first time. Additionally, we present calculations of the band structures and elastic and dynamic properties, which explain the nature of the structural rearrangement in the EuYCuTe_3_ crystals as a second-order phase transition. Moreover, we model a monolayer peeling and study its dynamic properties theoretically.

## 2. Materials and Methods

### 2.1. Materials

The elements Eu (99.99%), Y (99.99%), Te (99.9%), Ar (99.99%) as well as C_2_H_2_ (99.9%) and CsI (99.9%) were purchased from ChemPur (Karlsruhe, Germany), while Cu (99.999%) was obtained from Aldrich (Milwaukee, WI, USA).

### 2.2. Synthesis

The starting compounds were taken in the ratio of 1 Eu:1 Cu:1 Y:3 Te. To obtain the chalcogenide EuYCuTe_3_ with a mass of 0.25 g, the following amounts of the initial components were used: *m*(Y) = 0.0323 g, *m*(Cu) = 0.0231 g, *m*(Eu) = 0.0553 g, *m*(Te) = 0.1393 g. CsI was taken in a threefold excess relative to the total mass of all starting compounds.

Elementary copper, tellurium, europium and yttrium were crushed into small pieces measuring 2–3 mm in an agate mortar, weighed on analytical scales, and placed in a glassy silica ampoule. To lower the reaction temperature, 800 mg of cesium iodide were added as a fluxing agent. All operations were conducted in an argon atmosphere of a glove box. A layer of amorphous carbon, obtained by acetylene pyrolysis, was preliminarily applied to the inner walls of the silica ampoules. The ampoules were hermetically sealed with a quick fit, removed from the glove box, evacuated to 2·10^−3^ mbar, torch sealed, placed in a muffle furnace, heated to 1120 K for 30 h, maintained for 96 h, and then cooled to 570 K over 140 h and finally down to room temperature over 3 h. The reaction proceeded as follows: Eu + Cu + Y + 3 Te ⟶ EuYCuTe_3_. CsI did not chemically interact with the starting compounds or reaction products. The crude product was purified from the residual flux using demineralized water. The product consisted entirely of black, needle-like crystals of EuYCuTe_3_ up to 500 µm in size, and no by-products could be observed. To obtain the second polymorph of EuYCuTe_3_ with the space group *Cmcm*, the obtained crystals were additionally heated to 323(2) K, with no change in crystal color. The obtained crystals were suitable for single crystal X-ray diffraction analysis and taking measurements of their magnetic and optical properties. Unfortunately, high-quality powder X-ray diffraction patterns could not be obtained, as copper compounds are strong absorbers of molybdenum radiation.

### 2.3. X-Ray Diffraction Analysis

The single-crystal diffraction intensities for the *Pnma*-form of EuYCuTe_3_ were obtained at room temperature 293(2) K, while for the *Cmcm*-form of EuYCuTe_3_ the data collection temperature was 323(2) K using a *κ*-CCD single crystal diffractometer (Bruker AXS, Billerica, MA, USA) equipped with a CCD detector, a graphite monochromator and a Mo-*K*_α_ radiation source. The unit–cell parameters were determined and refined for a set of 11880 and 6391 reflections for the *Pnma*- and the *Cmcm*-form, respectively. The unit-cell parameters of both modifications corresponded to the orthorhombic crystal system. The space groups (*Pnma* vs. *Cmcm*) were determined from the statistical analysis of the intensities of all reflections. Absorption corrections were applied using the program SADABS (2008). The crystal structure was solved by direct methods using the program SHELXS (2013) and refined in the anisotropic approximation using the program SHELXL (2013) [31]. Structural studies for missing symmetry elements were carried out using the program PLATON (2009) [32]. The crystallographic data have been deposited with the Cambridge Crystallographic Data Centre. The data can be downloaded from www.ccdc.cam.ac.uk/data_request/cif (accessed on 25 December 2024).

### 2.4. Magnetic Measurements

The magnetic measurements were performed via the MPMS3 measurement system by Quantum Design (San Diego, CA, USA). Using a SQUID magnetometer, the temperature dependencies of the magnetic moments of the EuYCuTe_3_ sample with a mass of 0.01473 g were measured in the temperature range from 2 to 300 K under constant external field of 40 kA∙m^−1^. Measurements were conducted in both FC (field-cooled) and ZFC (zero-field-cooled) modes. Another experiment, which consisted of measuring the dependence of the magnetic moment of the same sample, stayed at a constant temperature on changing the external magnetic field using a vibrating sample magnetometer. The field varied from 0 to ±4 MA∙m^−1^ in magnitude; the temperature points were 2 and 300 K.

### 2.5. Spectroscopy of the Raman Scattering

Raman spectra of the single crystal sample of EuYCuTe_3_ were acquired using a Horiba XploRa spectrometer (HORIBA Scientific, Kyoto, Japan). The excitation light at a wavelength of 532 nm was used. The acquisition conditions were as follows: filter-10, hole-300, slit-100, and resolution-2400.

### 2.6. DFT Calculations

The structure and properties of *α*- and *β*-EuYCuTe_3_ crystals were calculated within the framework of the DFT approach for the first time.

The structure and dynamics of the crystal lattice were calculated using a *pseudo*-potential «4f in core» for the rare-earth ion Eu^2+^ and a valence basis set to describe the outer electrons 5*s*^2^5*p*^6^ that are involved in the chemical bond. This approach allows an adequate description of the crystal structure, phonon spectrum, and elastic properties.

The structure and dynamics of the crystal lattice were calculated by using hybrid B3LYP functional. Thus, the contribution of non-local *Hartree-Fock* exchange was taken into account. The calculations have been performed in a program CRYSTAL17 [33,34] designed for modeling periodic structures within the framework of the MO LCAO approach.

The electronic band structure and magnetic moments ordering were calculated with explicit consideration of 4f shell of europium in the plane wave basis at the level GGA+U (*U* = 4.5 eV) using ABINIT v9.8 software package [35].

Details of calculations of the structure and dynamics of the crystal lattice in CRYSTAL program. The *Pseudo*-potential ECP*53*MWB with attached basis set of TZVP type have been used for europium Eu^2+^ [33,36,37]. That is, this potential replaces the electron shells of europium, including the 4*f* shell. The outer shells 5*s*^2^5*p*^6^, which form the chemical bond, were described by means of wave functions. This *pseudo*-potential and valent basis set is available on the University of Cologne website [38]. For yttrium *pseudo*-potential, «Y_POB_TZVP_2018» [34] was also used. This *pseudo*-potential replaces electron shells with (*n* = 1–3). The outer shells with (*n* = 4–5) were described by means of a valent basis set of the TZVP type. For copper ions, we used a full-electron basis set «Cu_86-4111(41D)G_doll_2000» [34]. For tellurium, we also used a full-electron basis set [39]. In the tenth shell of tellurium, the exponent was set to 0.17. *Gaussians* whose exponents are less than 0.1 were deleted from the basis sets since the calculations are periodic.

The tolerance at calculations of two-electron integrals was at least 10^−8^ a. u. The tolerance at the calculation of the self-consistent field was 10^−9^ a.u. The *Monkhorst*-*Pack* scheme is used at CRYSTAL code for integration over the *Brillouin* zone. We used k-points mesh 8 × 8 × 8 for this.

Full optimization of the crystal structure has been performed for *α*- and *β*-EuYCuTe_3_ crystals, which include atomic positions and unit–cell parameter relaxation until forces on atoms become less than 10^−5^ Ha/Bohr and stress tensor components reach values below 0.01 GPa. Phonon spectra at the Gamma point were calculated for the optimized structure. Tensor of elastic constants was calculated for optimized crystal structure too. Details of the calculations are described in the work [40]. We used B3LYP functional [41]. It is a well-known hybrid functional with part of the *Hartree-Fock* exchange equal to 20%. B3LYP is a widely used DFT functional. A huge number of compounds with ionic and covalent bonds were described by using B3LYP [42].

The phonon-dispersion calculations were performed using a super-cell approach within the frozen phonon method as implemented in the “PHONOPY” software package (Version 2.18) [43]. The 2 × 2 × 2 size of super-cell was used for bulk phases and 2 × 2 × 1 for layered structures. The *Grüneisen* parameter was obtained by the calculation of phonon states calculated for a set of structures with various unit–cell volumes. The optimization of structural parameters with a fixed volume of the unit cell during exfoliation simulation was held using the BFGS algorithm as implemented in the atomic simulation environment “Python library” [44].

## 3. Results

### 3.1. Layered Crystal Structures of α- and β-EuYCuTe_3_

According to X-ray diffraction analysis of the single crystals, it has been established that the quaternary telluride EuYCuTe_3_ is dimorphic. After ampoule synthesis from elements taken in a ratio of 1 Eu:1 Y:1 Cu:3 Te, heated to 1120 K in excess with a CsI flux and slowly cooled to room temperature, orthorhombic crystals with *Pnma* symmetry of the structure type Eu_2_CuS_3_ were obtained. Crystallographic data, details of data collection, atomic coordinates, thermal displacement parameters, bond lengths and valence angles are presented in Table 1 and Table 2 and Tables S1 and S2 in the Supplementary Material. The obtained modification is designated as *α*-EuYCuTe_3_. The quaternary chalcogenides of the isostoichiometric composition EuYCuSe_3_ [27] and EuYCuS_3_ [8] are isostructural with the telluride. Heating the obtained single crystals of *α*-EuYCuTe_3_ to 323 K resulted in a change in symmetry of the orthorhombic crystals and the formation of a more symmetric structure in the space group *Cmcm* of the structure type KZrCuS_3_ (Table 1 and Table 2 and Tables S1 and S2 in the Supplementary Material). This modification is designated as *β*-EuYCuTe_3_. The parameters obtained from DFT calculations are *a* = 11.1669 Å, *b* = 4.3248 Å, *c* = 14.4700 Å for *α*-EuYCuTe_3_ and *a* = 4.3375 Å, *b* = 14.4812 Å, *c* = 11.1074 Å for *β*-EuYCuTe_3_ and, thus, are in good agreement with those determined experimentally (Table 1).

**Figure 1:**
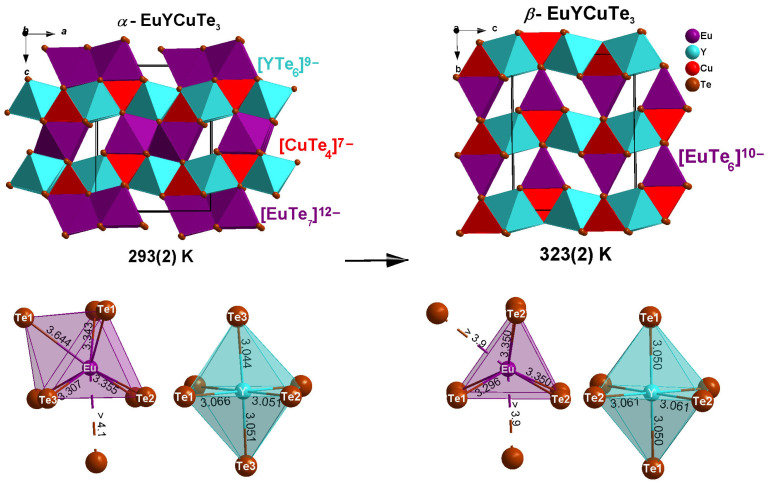
View at the orthorhombic crystal structures of *α*- and *β*-EuYCuTe_3_ with the space groups *Pmma* (left view) and *Cmcm* (right view) along the *b*- and the *a*-axis, respectively, together with the telluride coordination polyhedra formed around metal cations.

The crystal structures of both orthorhombic polymorphs of EuYCuTe_3_ exhibit similarities and differences (Figure 1). The similarity lies in the fact that in both modifications (*α* and *β*), the cations Eu^2+^, Y^3+^ and Cu^+^ occupy independent crystallographic positions. Both structures contain distorted vertex-connected [CuTe_4_]^7−^ tetrahedra, which form chains along the [010] direction in *α*-EuYCuTe_3_ and along the [100] direction in *β*-EuYCuTe_3_. The Cu–Te bond lengths *d*(Cu–Te) are in the ranges of 2.634(1)–2.673(1) Å (*α*-form) and 2.645(2)–2.676(2) Å (*β*-form), which appear lower than the theoretical value of 2.81 Å [22]. The values of the bond angles ∡(Te–Cu–Te) deviate from the ideal tetrahedral angle by 2.9–4.6% (*α*-form) and 1.7–4.6% (*β*-form) (Table 2). The degree of distortion of the tetrahedra decreases, when transitioning from the *α*- into the *β*-modification. The descriptor values τ_4_ = 0.96 in *α*-EuYCuTe_3_ and τ_4_ = 0.98 in *β*-EuYCuTe_3_ indicate 13–27% distortion from the ideal tetrahedron towards a trigonal pyramidal shape [45]. In both structures, the Y^3+^ cations reside in sixfold coordination with the distorted [YTe_6_]^9−^ octahedra, forming layers by edge connection. The bond angles ∡(Te–Y–Te) deviate from the ideal octahedral angle (Table 2). The tetrahedral and octahedra form parallel two-dimensional layers in the (110) or (101) planes, respectively. The main difference between the *α*- and *β*-form structures results from the coordination of the Eu^2+^ cations, however. In the structure of *α*-EuYCuTe_3_, they are centers of a monocapped trigonal prismatic coordination sphere, forming chains along the [010] direction by face connection, with coordination polyhedra connected in pairs. In the structure of *β*-EuYCuTe_3_, they adopt a trigonal prismatic coordination, forming analogous chains along the [100] direction. If one considers two far away Te^2−^ anions at a distance of 3.900 Å (2×) as coordinative relevant by adding two caps (Te2’) to the rectangular faces of the [EuTe_6_]^10−^ prisms, an extended sphere [EuTe_6+2_]^14−^ would result. But being 17.1% further apart then the mean Eu–Te value of 3.332 Å, the sums of the valence efforts considering coordination are Eu (1.90), Y (3.01), Cu (1.40) in *α*-EuYCuTe_3_ and Eu (1.63), Y (2.99), Cu (1.39) in *β*-EuYCuTe_3_. There is also one more far away Te^2−^ ligand for Eu^2+^ in *α*-EuYCuTe_3_, but this one (Te2’) has a distance of 4.156 Å and should not count for a [EuTe_6+1+1_]^14−^ polyhedron in this case.

Thus, the three-dimensional crystal structures of both polymorphs of EuYCuTe_3_ are formed by two-dimensional layers consisting of tetrahedra and octahedra in the *ab* (*α*-form) and *bc* planes (*β*-form), separated by chains of monocapped trigonal prisms (*α*-form) and trigonal prisms (*β*-form).

As the radius of the chalcogenide anions increases in the series of yttrium compounds EuYCu*Ch*_3_ (*Ch* = S [8], Se [27], Te), which crystallize in the space group *Pnma*, a decrease in the τ_4_ descriptor value is observed: 0.984 [8] → 0.976 [27] → 0.958 (this work), and the distortion degree of the [Cu*Ch*_4_]^7−^ tetrahedral increases by up to 13%. The parameters and volume of the unit cell also increase as follows:

*a* = 10.1830(3) Å (*Ch* = S) [8] → 10.566(1) Å (*Ch* = Se) [27] → 11.2730(7) Å (*Ch* = Te) (Table 1);
*b* = 3.9260(1) Å (*Ch* = S) [8] → 4.0800(5) Å (*Ch* = Se) [27] → 4.3214(3) Å (*Ch* = Te) (Table 1);
*c* = 12.8482(4) Å (*Ch* = S) [8] → 13.401(2) Å (*Ch* = Se) [27] → 14.3271(9) Å (*Ch* = Te) (Table 1);
*V* = 513.7(1) Å^3^ (*Ch* = S) [8] → 577.7(1) Å^3^ (*Ch* = Se) [27] → 697.95(8) Å^3^ (*Ch* = Te) (Table 1).

In the series of chalcogenides EuYCu*Ch*_3_ (*Ch* = S [8], Se [27], Te), which crystallizes with the space group *Pnma*, a systematic increase in the average metal-chalcogen bond lengths is also observed, according to:

*d*(Eu–*Ch*): 3.029 Å [8] → 3.147 Å [27] → 3.379 Å (this work);
*d*(Y–*Ch*): 2.752 Å [8] → 2.856 Å [27] → 3.057 Å (this work);
*d*(Cu–*Ch*): 2.350 Å [8] → 2.481 Å [27] → 2.659 Å (this work).

No polymorphic modifications have been found in the quaternary yttrium sulfide and selenide EuYCu*Ch*_3_ (*Ch* = S, Se) thus far. The increase in the metal–chalcogen bond length may accelerate the transformation of the local geometry around the cations and lead to the emergence of a second polymorphic modification in the telluride EuYCuTe_3_ with a relatively small temperature change of 30 K. It was previously known as the telluride of the alkaline earth element BaYCuTe_3_ [7], which crystallizes in the space group *Cmcm*. Thus, in the yttrium telluride, the increase in the radius of the divalent metal, such as *r*_i_ (Ba^2+^) = 1.35 Å (with a coordination number of 6 [22]), is greater than *r*_i_(Eu^2+^) = 1.17 Å, and consequently, the increase in the bond length *d*(*M*^2+^–Te): 3.379 Å (*M*^2+^ = Eu^2+^) → 3.473 Å (*M*^2+^ = Ba^2+^) also stabilizes the *Cmcm*-form.

Therefore, for the first time in the family of quaternary chalcogenides *MRE*Cu*Ch*_3_, a polymorphic transition has been discovered that changes not only the structure type, but also the space group. Previously, only changes in structure types were observed in isomorphic compounds, while maintaining the same space group in areas of crystallochemical instability [12,14,24,46,47].

### 3.2. Magnetic Properties of EuYCuTe_3_

**Figure 2:**
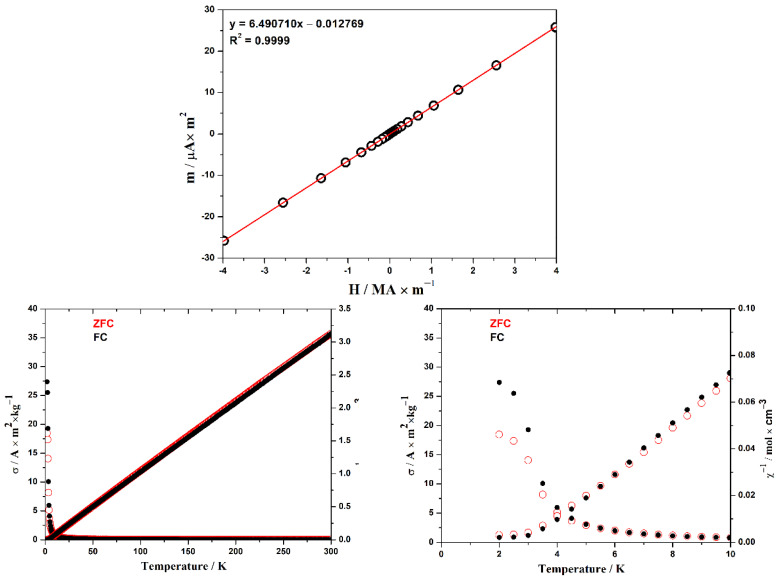
Top: magnetic moment of EuYCuTe_3_ at 300 K versus external field. Bottom: specific magnetization (left ordinate) and reciprocal molar susceptibility (right ordinate) versus temperature. Bottom right: the same at low temperatures. Black dots—FC mode, red circles—ZFC mode.

Experimental data on the magnetic properties of the EuYCuTe_3_ sample contain the temperature dependence of magnetization at a constant external field and the field dependence at a constant temperature. These dependencies are pictured in Figure 2. The value of the constant magnetic field is 39.8 kA∙m^−1^, and the constant temperature amounts to 300 K. This figure also shows the temperature dependence of the inverse molar magnetic susceptibility calculated from primary experimental data.

The temperature dependence of the inverse susceptibility above 50 K is well described by the *Curie–Weiss* law ($\chi^{-1}=\frac{T-\theta_{p}}{C}$) with the following parameter values: *C* = 0.095 K∙m^3^∙kmol^−1^, *θ*_p_ = 1.5 K. Here, *C* is the *Curie* constant, and *θ*p is the paramagnetic *Curie* temperature. The corresponding effective magnetic moment of *μ* = 7.77 *μ*_B_ was also calculated for this constant. Deviations of the experimental points from the *Curie* law with these parameters do not exceed 1%. The value of the *Curie* constant and the effective moment are close to those calculated in the theory of paramagnetism for non-interacting Eu^2+^ cations: 0.0990 K∙m^3^∙kmol^−1^ and 7.937 *μ*_B_. The positive sign of *θ*_p_ indicates a predominantly ferromagnetic nature of the interaction of Eu^2+^ magnetic moments in the paramagnetic region. Below 3 K, the curves for the FC and ZFC modes diverge, indicating a transition to a ferromagnetic state. A decrease in the phase-transition temperature is observed in the series of yttrium chalcogenides: 4.5 K for EuYCuS_3_ [8], 3.3 K for EuYCuSe_3_ [27] and 3.0 K for EuYCuTe_3_.

The field dependence of the magnetic moment at 300 K is precisely linear. This indicates that the sample is in a paramagnetic state. From the *Curie* law for paramagnets (*m* = *HC*/*T*), the *Curie* constant and the corresponding effective magnetic moment were estimated to be *C* = 0.091 K∙m^3^∙kmol^−1^ and *μ* = 7.60 *μ*_B_, which expectedly gives slightly underrated values as compared to those calculated from the temperature dependence of the susceptibility.

**Figure 3:**
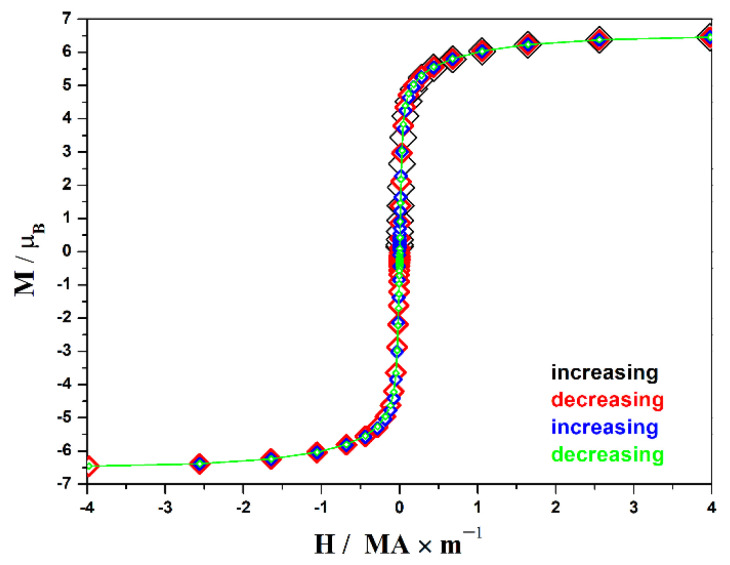
Magnetization curves of EuYCuTe_3_ at 2 K.

The appearance of the field dependence graph at 2 K (Figure 3) indicates a ferromagnetic state of the sample. The hysteresis loop width is less than 4 kA∙m^−1^, which is typical for soft ferromagnets. Saturation begins around 0.4 MA ∙ m^−1^, with the magnetic moment per formula unit in a maximum field of 4 MA ∙ m^−1^ reaching 6.5 *μ*_B_, compared to the theoretical value *g*J = 7 for Eu^2+^ cations, which determine the magnetic properties of the compound.

### 3.3. Raman, IR and Phonon Spectra

**Figure 4:**
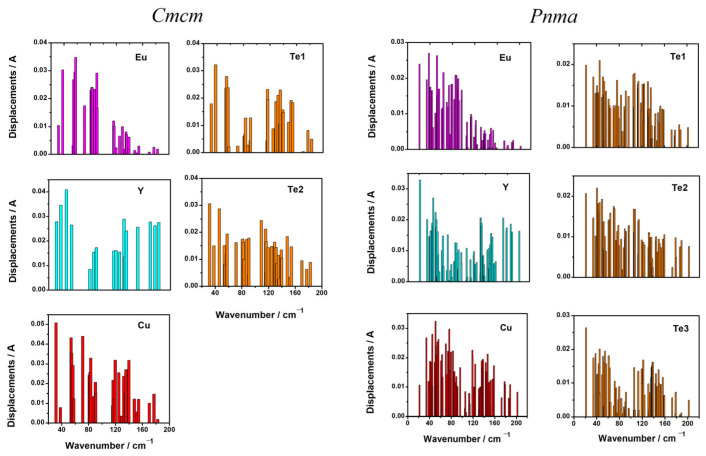
The displacement of ions at phonon modes in EuYCuTe_3_, (*Cmcm*-form, left and *Pnma*-form, right).

**Figure 5:**
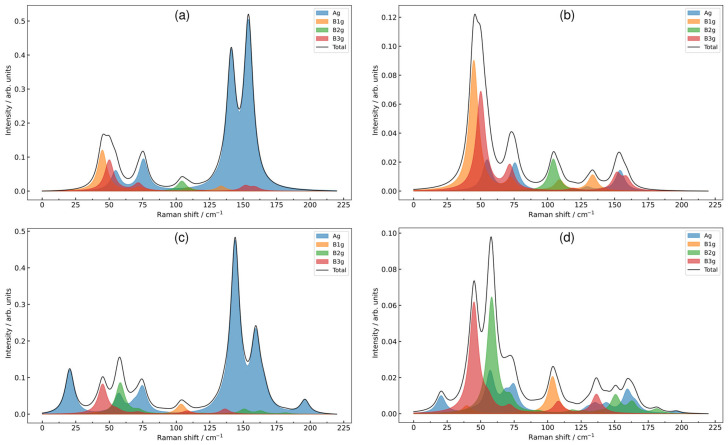
Simulated EuYCuTe_3_ Raman spectra of the *Cmcm* (**a**,**b**) and the *Pnma* (**c**,**d**) phase in parallel (**a**,**c**) and crossed (**b**,**d**) polarization setups of powder sample. The black lines denote total intensity and colored filled areas show the contribution of vibrational modes with Ag (blue), B1g (orange), B2g (green) and B3g (maroon) irreducible representations.

After optimization of the crystal structure, the phonon spectrum was calculated at the Γ-point (Tables S3 and S4 in the Supplementary Material). From the analysis of the displacement vectors obtained in the ab initio calculation, the displacements of the ions in each phonon mode were determined (Figure 4). The results of modeling the Raman spectrum for both forms (*Pnma* and *Cmcm*) of EuYCuTe_3_ are shown in Figure 5, when EuYCuTe_3_ crystallizes in the *Cmcm* structure for *Z* = 4. Accordingly, the number of phonon modes in the *Pnma* structure is twice as much. EuYCuTe_3_ has 15 Raman modes in the *Cmcm* structure and 36 modes in the *Pnma* structure. Accordingly, the Raman spectra of these structures differ significantly, as can be seen in the DFT-calculated spectra plotted in Figure 5. In order to distinguish the nature of spectra evolution, the decomposition of total spectra on phonon species was carried out. Namely, the impact or A_g_ modes in the Raman spectrum is highlighted with a blue-filled area; hence, one may find that an extra band in the low-frequency range in the *Pnma* phase is attributed to Ag states. The bands attributed to B_1g_ and B_2g_ modes are highlighted by orange and green color, respectively, and considerable influence on spectra in the crossed polarization setup. The bands related to B_3g_ phonon states are highlighted by maroon color in the spectra and give a considerable impact in low frequency ranges.

**Figure 6:**
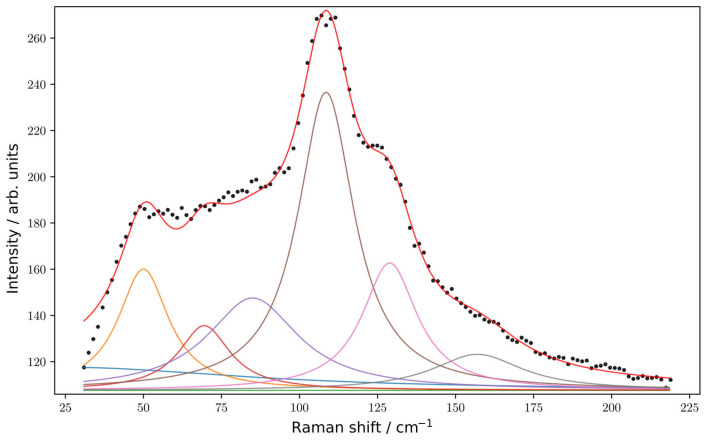
The experimental Raman spectrum (black markers) of EuYCuTe_3_ in random orientation decomposed by Lorenzian fitting on corresponding vibrational bands (color lines). Red line denotes summary fitting.

The frequencies of phonons in both the *Pnma* phase and the *Cmcm* phase do not exceed 200 cm^−1^. In such a low-frequency range, the participation of almost all ions is manifested simultaneously (Tables S3 and S4 in the Supplementary Material, column «participating ions»). The experimental Raman spectra are plotted in Figure 6. One can find a fair agreement between the calculated Raman spectrum for the *Pnma* phase and the experimental one. The calculated spectrum of the *Pnma* phase reproduces very well the doublet in the vicinity of 150 cm^−1^, namely, the low-frequency band is most intense in contrast to the spectrum calculated for the high symmetry *Cmcm* phase.

**Figure 7:**
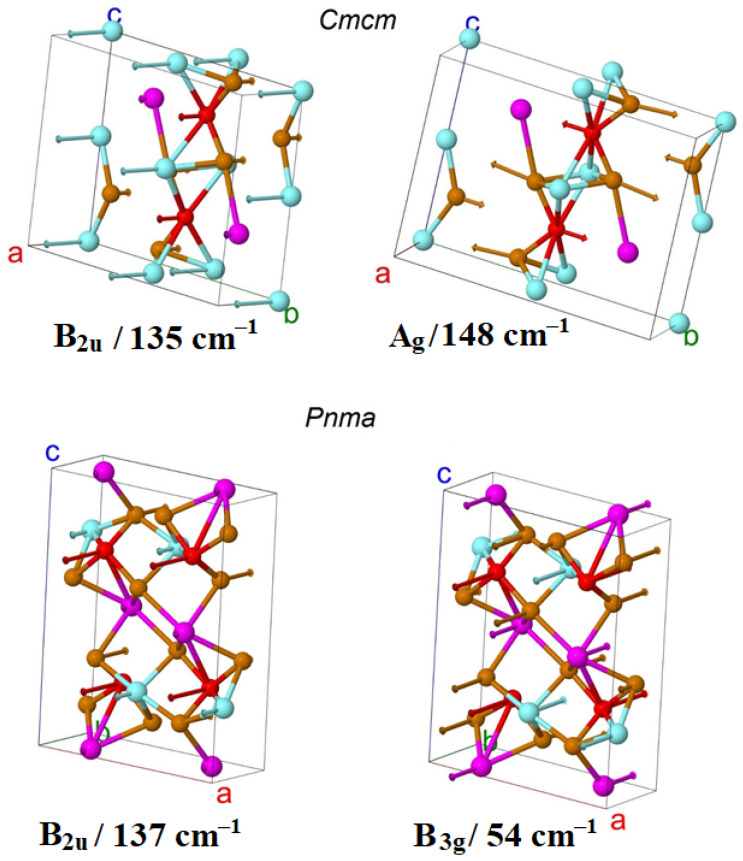
Ion displacements in two IR and Raman modes with maximum intensity in EuYCuTe_3_ (*Cmcm*-form, top, and *Pnma*-form, bottom). The *a*, *b*, *c* legends denotes lattice parameters.

A strong mixing of structure units vibrations can be noted. In the *Cmcm* phase, europium is strongly involved in modes with frequencies up to 90 cm^−1^, copper up to 140 cm^−1^, and tellurium up to 150 cm^−1^. Yttrium is involved in the entire frequency range (Figure 4). The calculation predicts that the phonon spectrum of the *Cmcm* phase has a gap of about 60–70 cm^−1^, as well as a gap of about 90–110 cm^−1^. In the *Pnma* phase, europium is strongly involved in modes with frequencies up to 90 cm^−1^, copper up to 160 cm^−1^, and tellurium up to 150 cm^−1^. Yttrium is involved in the entire frequency range as in the *Pnma* phase. There are significantly more phonon modes in this phase, and the gaps in the phonon spectrum are significantly narrower. The calculation predicts that the phonon spectrum of the *Pnma* phase has a gap of about 96–105 cm^−1^, as well as a gap of about 160–170 cm^−1^. Note that the gaps in the phonon spectrum of the *Pnma* phase lie in approximately the same range as those of the *Cmcm* phase. This indicates oscillations of similar structural units. The calculation predicts that in most modes, all or almost all ions participate. However, several modes can be distinguished in which ions of only one or two types participate. For example, in the phase *Cmcm,* only Te2 ions participate in the B_3g_ mode (109 cm^−1^). In the high-frequency mode B_3u_ (183 cm^−1^), yttrium ions are predominantly involved. In the *Pnma* phase, Te1 and Te2 ions are predominantly involved in mode B_2u_ mode (105 cm^−1^). In the high-frequency mode B_2g_ (202 cm^−1^), yttrium ions are predominantly involved. Figure 7 shows the ion displacements in the most intense Raman mode and in the most intense IR mode in the *Cmcm* and *Pnma* phases, respectively.

### 3.4. Band Structure of EuYCuTe3

**Figure 8:**
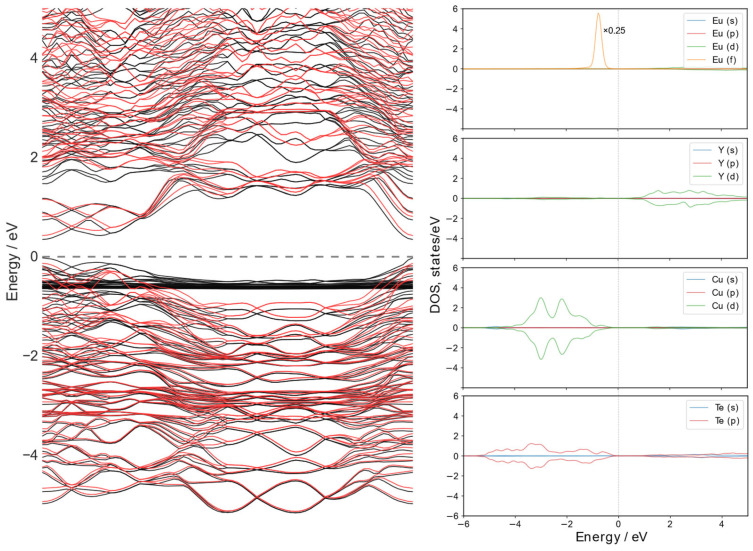
The calculated in DFT+U approximation band structure (**left**) and projected on atomic species density of states (**right**) for the *Pnma* phase of EuYCuTe_3_. The energy level is shifted by *Fermi* energy for the sake of clarity. The black and red lines in band structure are branches with spin up and down states respectively.

The electronic structure of EuYCuTe_3_ is studied by calculations of band structure and projected density of states. The electronic states are calculated along the path in the *Brillouin* zone through the most highly symmetric points for the orthorhombic lattice. For the *Pnma* phase the path is composed as Γ-X-Z-U-Y-S-T-R-Γ. The coordinates of these points are (0, 0, 0), (^1^/_2_, 0, 0), (0, 0, ^1^/_2_), (^1^/_2_, 0, ^1^/_2_), (0, ^1^/_2_, 0), (^1^/_2_, ^1^/_2_, 0), (0, ^1^/_2_, ^1^/_2_), (^1^/_2_, ^1^/_2_, ^1^/_2_), (0, 0, 0), respectively. For the *Cmcm* phase, the path goes through Γ-Y-T-Z-S-R-Γ. The coordinates of these points are (0, 0, 0), (^1^/_2_, ^1^/_2_, 0), (^1^/_2_, ^1^/_2_, ^1^/_2_), (0, 0, ^1^/_2_), (0, ^1^/_2_, 0), (0, ^1^/_2_, ^1^/_2_), and (0, 0, 0), respectively. The band structure of the *Pnma* phase is plotted in Figure 8. The spin-up states are plotted with black lines, while the spin-down states are denoted with red. The strong magnetic ordering can be clearly seen as up and down states significantly differ (Figure 8, left). According to calculations of projected density of states (PDOS) shown in Figure 8 (right), the top of the valence band is formed mainly by the states of copper and tellurium, and the bottom of the conduction band is formed by *d*-states of yttrium atoms. It is noteworthy that 4*f*-states of europium atoms are localized in the vicinity of the valence band top. These states show a very low dispersion and, therefore, do not take part in chemical bond compositions, which is very common for highly correlated *f*-states. However, several branches attributed to 4*f*-states of rare-earth elements show strong dispersion in the vicinity of the Γ-point of BZ and can contribute to the optical transition process. The band gap is defined as the difference in energy between the top of the valence band and the bottom of the conduction band. According to calculations, for the *Pnma* phase of the EuYCuTe_3,_ it is an ultra-narrow semiconductor with direct optical transition and a value of the band–gap equal to *E*_g_ = 0.46 eV. It should be noted that the well-known DFT problem in the estimation of the band–gap value can be partially resolved by applying a *Habbard* correction to density functional (which is the case in our calculations), but still can suffer from complete improvement to the problem. Therefore, additional calculations of the band–gap value were performed using the hybrid B3LYP functional, which predicted an *E*_g_ value equal to 1.86 eV. Such underestimation can be explained in part by replacements of valence 4*f*-states in core ones during *pseudo*-potential construction. Keeping in mind that the samples are deeply dark with a metallic lustre, one may assume that the actual value of *E*_g_ is in the range of calculated values closer to the lower one.

The calculated band structure of the *Cmcm* phase is plotted in Figure S1 (Supplementary Material). This demonstrates an indirect fundamental band gap with the highest energy electronic states in the valence band at the Γ-point and the lowest energy of the conduction band at the Y-point of BZ. Thus, the high-temperature phase is a semiconductor with an indirect fundamental band gap. The value of *E*_g_ equal 0.4 eV and 1.76 eV in DFT+U and B3LYP approximations, correspondingly.

The influence of magnetic order on europium atoms on the electronic structure was studied as well in the assumption of collinear magnetism for the low-temperature *Pnma* phase. There are four magnetic atoms in the unit cell, since europium takes *4c Wyckoff* positions with coordinates (x, ^1^/_4_, z), (–x + ^1^/_2_, ^3^/_4_, z + ^1^/_2_), (–x, ^3^/_4_, –z), (x + ^1^/_2_, ^1^/_4_, –z + ^1^/_2_). Therefore, a number of non-equivalent distributions of spins in the unit cell is possible. Namely, a ferromagnetic state F: [↑↑↑↑] (all magnetic moments are collinear) and three general antiferromagnetic orderings (AF1: [↓↑↑↓]; AF2: [↑↓↑↓]; AF3: [↑↑↓↓]). All other combinations are either degenerated with the above-mentioned ones or failed to converge. The calculated total energies for these configurations reveal the ferromagnetic state as the most stable one with the lowest energy. The difference of total energy per unit cell with respect to ferromagnetic ordering is equal to 1.12, 2.83 and 1.88 meV for the AF1, AF2 and AF3 configurations, respectively. The calculated value of magnetic moment per formula unit in case of the most stable ferromagnetic ordering is equivalent to 6.89 μ_B_. The calculated value perfectly matches the experimental data reported in the section “Magnetic Properties of EuYCuTe_3_”.

### 3.5. Phonon Dispersion

**Figure 9:**
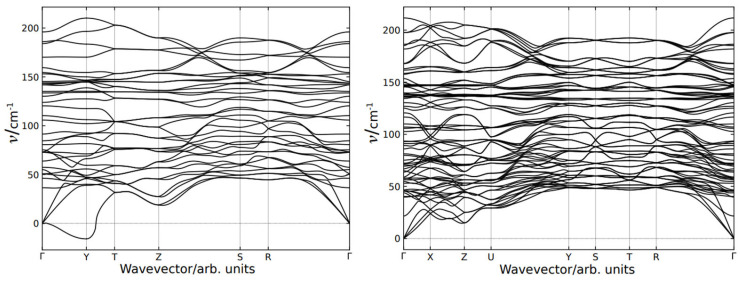
Dispersion of the phonon branches of EuYCuTe_3_ in the high-symmetry (*Cmcm*, **left**) and the low-symmetry case (*Pnma*, **right**).

The calculated phonon dispersion branches of EuYCuTe_3_ are plotted in Figure 9 for both the *Cmcm*- and the *Pnma*-form. The high-symmetry points are labeled according to paper [48]. It is noteworthy that the number of branches is twice as much in the case of the *Cmcm* phase with respect to the *Pnma* phase due to the difference in a number of atoms in the unit cell with a factor of two. Although the number of branches is different, the dispersion for these phases appears alike. As can be seen in Figure 9 (right), the branches are nearly degenerated; thus, the dispersion of the *Pnma* phase is formed by coupled branches. Therefore, the phonon states in these phases are very close, except for the lowest frequency acoustical branch of the *Cmcm* phase, which demonstrates the extraordinary behavior in the vicinity of the Y-point (−^1^/_2_, ^1^/_2_, 0) of the *Brillouin* zone (BZ), as shown in Figure 9 (left).

**Figure 10:**
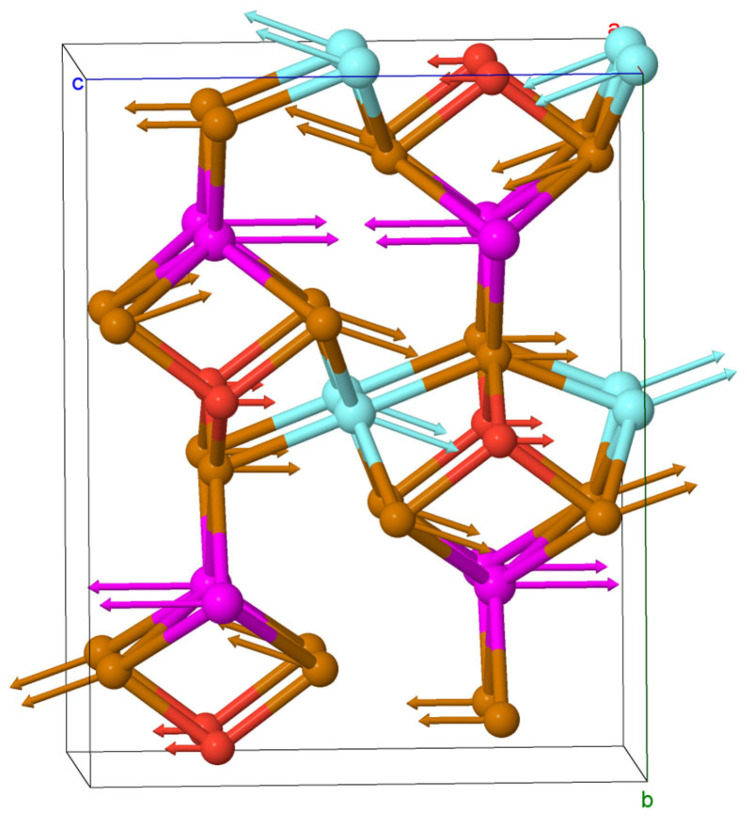
Atomic displacement pattern of the imaginary mode in the Y-point of the *Brillouin* zone in *Cmcm*-form of EuYCuTe_3_. Brown, red and magenta circles denote Te, Cu and Eu atoms, respectively, for the cyan circles correspond to Y atoms.

The mode with imaginary frequency is obtained in the Y point of the base-centered phase, which indicates on structural instability with respect to phonon states. This mode corresponds to *anti*-phase displacement of atoms in neighborhood layers perpendicular to the *b*-axis as plotted in Figure 10. As will be shown later, the bulk crystal can be exfoliated on these layers. It is noteworthy that all atoms are displaced in this unstable mode mostly along the *c*-axis.

As soon as the calculations are held in harmonic approximation, which is equivalent to experimental conditions at zero temperature the appearance of the imaginary mode reveals an instability of the *Cmcm* phase at low temperatures. Therefore, the experimentally observed structural phase transition is driven by condensation of the soft mode at the Y-point of the *Brillouin* zone (BZ) and accompanied by Y→ Γ (BZ) folding.

According to symmetry analysis, the transformation matrix of the high-symmetry phase basis to the low-symmetry one equals to:

(1) $$Tr=\left(\begin{array}{ccc} 0 & 1 & 0\\ 0 & 0 & 1\\ 1 & 0 & 0 \end{array}\right)\left(\begin{array}{c} 1/4\\ 1/4\\ 0 \end{array}\right)$$

**Figure 11:**
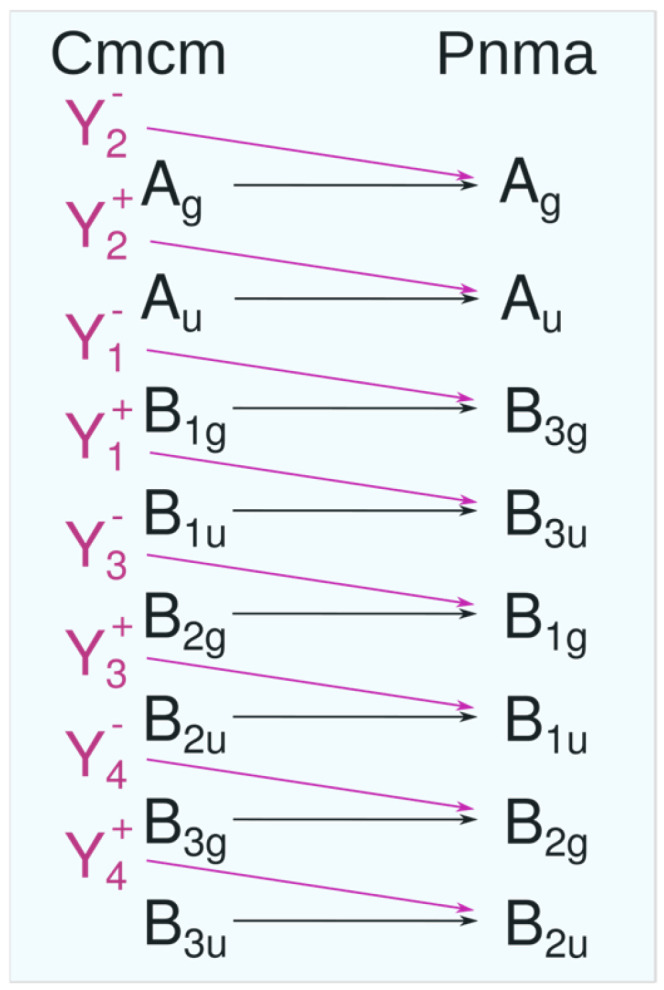
Correlation diagram between the irreducible space-group representations for the parent high-symmetry (*Cmcm*) and low-symmetry phase (*Pnma*).

The irreducible representation of the soft mode is Y_2_^−^; thus, using the program CORREL [49] of the Bilbao Crystallographic Server (BCS), one obtains the correlation diagram for IRreps between the *Cmcm* and *Pnma* phases, as shown in Figure 11.

According to this correlation diagram, the soft Y_2_^−^ mode of the *Cmcm* phase transforms into the A_g_ mode as a result of structural phase transition. The mode is active in Raman spectra of the low-symmetry *Pnma* phase, but the bands are less intensive related to other vibrational modes that are genetically connected with modes in Γ-point of the high-symmetry *Cmcm* phase (Figure 5).

The interest to the family of copper rare-earth metal chalcogenides in technical application as a key elements of efficient thermal energy management devices require estimation of its thermodynamic properties. The heat capacity at constant volume was calculated for EuYCuTe_3_ as a derivative of harmonic phonon energy with respect to temperature as implemented in the phonopy package [44]. The temperature dependence of heat capacity per one formula unit is plotted in Figure S2. The dependence is fitted within the *Debye* model, and the estimated *Debye* temperature is equal to *T*_D_ = 204 K for EuYCuTe_3_. The important parameter, which characterizes dynamical properties of a solid, is *Grüneisen* parameter *γ*, which is computed for each vibrational mode according to the following equation:

(2) $$\gamma_{n}=\frac{-V}{\omega_{n}}\frac{\partial \omega_{n}}{\partial V}$$

where *ω_n_* is the frequency of the *n*^th^ phonon mode and *V* is a volume of unit cell. The mean value is found to be equal to 1.4, which is close to other quaternary tellurides like BaCuScTe_3_ or CsCdYTe_3_ [5].

In order to approximately estimate the magnitude of lattice thermal conductivity, one may consider empirical equation, which show a very reasonable values for a big set of different compounds [50]

(3) $$\kappa_{L}=A\frac{ΜT_{D}^{3}\delta }{\gamma^{2}N^{2/3}T}$$

where *N* is number of atoms in the unit cell, *δ*^3^ is the volume per atom, *M* is the mean molar mass of formula unit and the empirical parameter *A* is given by the equation:

(4) $$A=\frac{2.43\cdot 10^{-8}}{1-0.514/\gamma +0.228/\gamma^{2}}$$

The value of thermal lattice conductivity obtained using Equation (3) is equal to 1.8 W/mK, several times less as compared to sodium chloride, and of the same value as reported for other quaternary tellurides [5]. Therefore, EuYCuTe_3_ can be classified as a low thermal conductivity material.

### 3.6. Elastic Constants and Elastic Modulus

**Figure 12:**
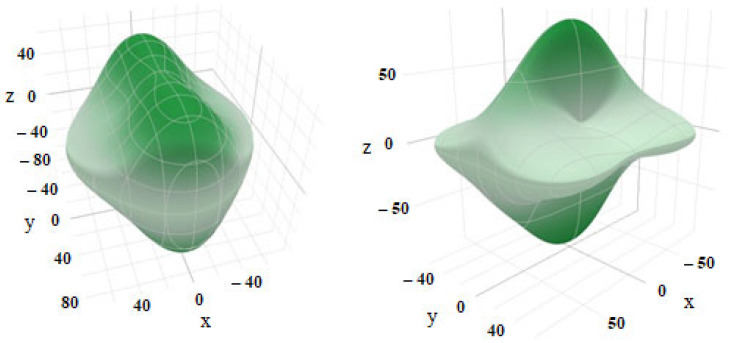
Dependence of the *Young’s* modulus (GPa) on the direction in the crystal structure of EuYCuTe_3_ (*Pnma* phase, **left** and *Cmcm* phase, **right**).

The results of the calculation of elastic constants and elastic moduli of EuYCuTe_3_ are given in Table 3. The *Born* stability criterion is performed for the tensor of elastic constants obtained from the calculation. The dependence of the *Young’s* modulus on the direction shows a significant anisotropy of the elastic properties (Figure 12). The anisotropy of elastic properties of EuYCuTe_3_ in the *Cmcm* phase is significantly greater than in the *Pnma* phase.

To estimate the anisotropy of the elastic properties of EuYCuTe_3_, we calculated the “universal” anisotropy index *A*^U^ according to [51]:

(5) $$A^{U}=5\left(\frac{G_{V}}{G_{R}}\right)+\frac{B_{V}}{B_{R}}-6,$$

where *B*_V_ and *B*_R_ are the bulk modules calculated in the *Voigt* and *Reuss* approximations, and *G*_V_ and *G*_R_ are the shear modules calculated in the *Voigt* and *Reuss* approximations, respectively. The deviation of the index *A^U^* (5) from zero determines the degree of anisotropic properties of the crystal. When the crystals of EuYCuTe_3_ have the *Cmcm* structure, the value of the anisotropy index *A^U^* is significantly greater (Table 3).

### 3.7. Layered Structure of EuYCuTe_3_

**Figure 13:**
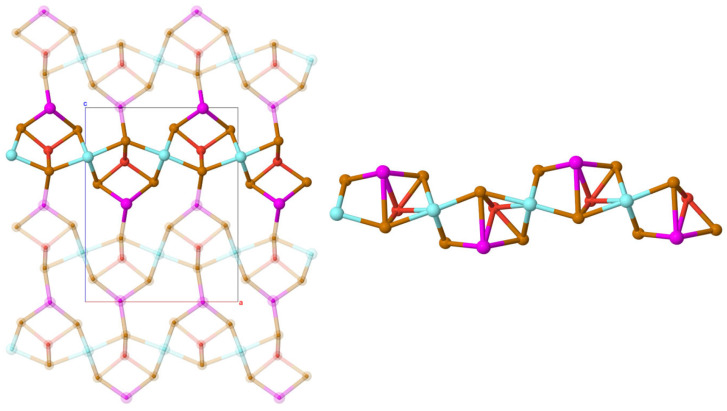
Visualization of a monolayer in the unit cell of bulk EuYCuTe_3_ (**left**) and relaxed structure of the monolayer structure (**right**).

In order to establish the possibility of exfoliation, the formation energy of the monolayer is theoretically studied. The simulation was held using step-by-step unit–cell expansion followed by optimization of the cell volume and the atomic positions with fixed volume of the unit cell. The structure in the *Pnma* phase was used as a starting point, and each optimization step was accompanied with 5% expansion of the unit–cell volume. When the expansion of the unit cell reaches 50%, the structure becomes clearly separated, and the symmetry of the layer is established as *pm2_1b* (no. 28) [52]. The obtained monolayer EuYCuTe_3_ structure is plotted in Figure 13 and the relaxed structural parameters are listed in Table 4. Compared to the bulk crystal, the unit cell of the layered structure is slightly expanded in the layer plane by about 6%, which is very common in case of layered structures [53]. At the same time, the layer is more compressed in the normal-to-layer direction, making the monolayer more flat due to the abrupt bonds between rare-earth metals and tellurium atoms.

The exfoliation energy for the 2D material is given by the expression [54]

*E*_exf_ = *E*_bulk_ − *E*_l_, (6)

where *E*_bulk_ and *E*_l_ are the total reduced energies (per atom) of the bulk crystal and optimized monolayer structure, respectively. The calculated value for the EuYCuTe_3_ exfoliation energy equals *E*_exf_ = −254 meV, which is several times higher than that of graphite (−50 meV) [55]. Therefore, the interlayer interaction in EuYCuTe_3_ does not purely consist of *van-der-Waals* interaction, but includes a covalent nature as well. The high value of the exfoliation energy also suggests an alternative to well-known strategies in the synthesis of monolayer structures, such as molecular beam epitaxy (MBE), chemical vapor deposition (CVD), atomic layer deposition (ALD), physical vapor deposition (PVD), or pulsed laser deposition (PLD).

**Figure 14:**
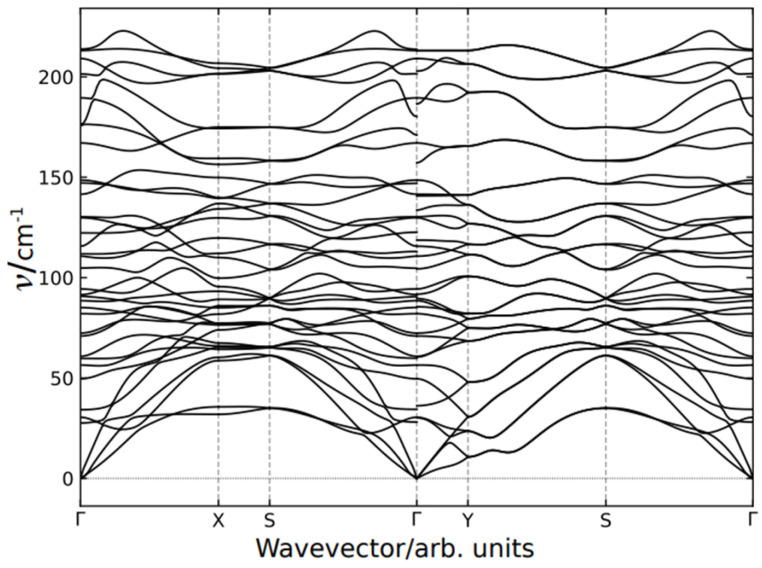
The monolayer EuYCuTe_3_ phonon-branch dispersion along high-symmetry points.

The stability of the theoretically predicted single-layer EuYCuTe_3_ structure is validated with respect to phonon states. The dispersion of vibration states was calculated and plotted in Figure 14. The coordinates of points in the *Brillouin* zone are X − (^1^/_2_ 0), Y − (0 ^1^/_2_), S − (^1^/_2_ ^1^/_2_) There are no vibrational modes with imaginary frequency values; therefore, the monolayer structure has a great potential for the real synthesis.

**Figure 15:**
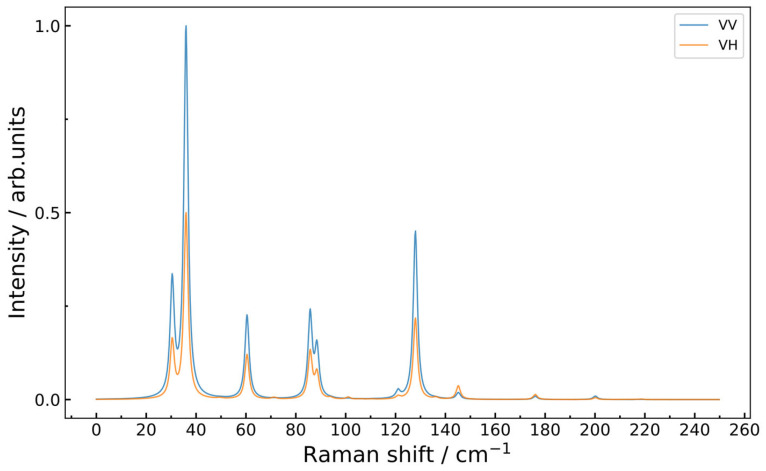
Raman spectrum simulated for a monolayered EuYCuTe_3_ structure in parallel (VV) and crossed (VH) polarizations.

The Raman scattering properties of the single-layer EuYCuTe_3_ structure are calculated, and the simulated Raman spectrum in different polarizations is plotted in Figure 15. It is noteworthy that the most intense band in the Raman spectrum can be found in the low-frequency range with a frequency of 36 cm^−1^, which corresponds to the a_1_ mode related to mixed valence Eu–Te and scissor Cu–Te–Cu vibrations. Hence, the band position in the spectra can be used to characterize local structure deformations due to substrate influence or defects in the structure.

## 4. Conclusions

In summary, we have successfully synthesized two polymorphic modifications of the layered telluride EuYCuTe_3_ using the halide-flux method. The crystal structures of the obtained polymorphs were determined using single-crystal X-ray diffraction. The orthorhombic crystals of EuYCuTe_3_ keep *Pnma* symmetry (*α*-EuYCuTe_3_, Eu_2_CuS_3_-type structure) at room temperature and exhibit a structural phase transition into the space group *Cmcm* (*β*-EuYCuTe_3_, KZrCuS_3_-type structure) at 323 K. The DFT calculations of dynamical and elastic properties reveal two distinct phases. Particularly, the anisotropy of the elastic properties in the *β* phase of EuYCuTe_3_ is significantly greater than in the *α* phase due to the decrease of [CuTe_4_]^7−^ tetrahedra distortion in the *Cmcm* phase, which leads to chain ordering along the crystallographic axes. The non-destructive method of phase determination based on peculiarities in Raman spectra calculated within DFT is developed. The imaginary mode is obtained in the high-symmetry *β* phase (*Cmcm*), and the structural phase transition *β*-EuYCuTe_3_ (*Cmcm*) → *α*-EuYCuTe_3_ (*Pnma*) is explained as the second-order phase transition, induced by soft-mode condensation at the Y-point of BZ, and is accompanied by doubling of the unit cell. Calculations of the electronic structure predict a direct band gap for the *α* phase and an indirect gap for the *β* phase. The monolayer configuration of EuYCuTe_3_ was obtained, and the value of exfoliation energy is found to be several times higher than that of graphite. Hence, the synthesis of 2D structures of EuYCuTe_3_ can be very different from well-known techniques. The stability of the monolayer structure is studied with respect to vibrational states, and the Raman spectra are simulated, where the most intensive band should be found in the low-frequency range. Finally, the quaternary europium(II) telluride EuYCuTe_3_ exhibits low-temperature ferromagnetic properties, and the obtained small value of the hysteresis loop in its magnetization curves allows us to characterize the layered structural chameleon EuYCuTe_3_ as a soft ferromagnet.

## Figures and Tables

**Table 1:** Main parameters of processing and refinement of the *α*- and *β*-EuYCuTe_3_ crystal data.

	*α*-EuYCuTe_3_	*β*-EuYCuTe_3_
Molecular weight (g/mol)	687.21	
Space group	*Pnma* (no. 62)	*Cmcm* (no. 63)
Structure type	Eu_2_CuS_3_	KZrCuS_3_
*Z*	4	
*a* (Å)	11.2730(7)	4.3231(3)
*b* (Å)	4.3214(3)	14.3328(9)
*c* (Å)	14.3271(9)	11.2843(7)
*V* (Å^3^)	697.95(8)	699.20(8)
*ρ*_cal_ (g/cm^3^)	6.540	6.528
*μ* (mm^−1^)	32.30	32.24
Reflections measured	14005	6391
Reflections independent	908	482
Reflections with *F*_o_ > 4*σ*(*F*_o_)	745	386
*θ*_max_ (◦)	27.38	27.45
*h*, *k*, *l* limits	±14, ±5, ±18	±5, ±18, ±14
*R*_int_	0.083	0.121
*Refinement results*		
Number of refinement parameters	38	24
*R*_1_ with *F*_o_ > 4*σ* (*F*_o_)	0.027	0.045
*wR*_2_	0.061	0.112
Goof	1.070	1.019
Δ*ρ*_max_ (e/Å^3^)	1.329	1.438
Δ*ρ*_min_ (e/Å^3^)	−1.278	−1.386
Extinction coefficient, *ε*	0.0023(1)	0.0026(2)
CSD-number	2355695	2355696

**Table 2:** Bond lengths (*d*/Å) and bond angles (∡/°) in the crystal structures of *α*- and *β*-EuYCuTe_3_.

*α*-EuYCuTe_3_					
**Bond lengths**					
Eu–Te3 ^i^	3.3075(7)	Y–Te3	3.044(1)	Cu–Te2	2.634(1)
Eu–Te3 ^ii^	3.3075(7)	Y–Te2 ^iv^	3.0509(8)	Cu–Te1	2.657(1)
Eu–Te1 ^iii^	3.3432(6)	Y–Te2 ^iii^	3.0509(8)	Cu–Te3 ^ii^	2.673(1)
Eu–Te1 ^iv^	3.3432(6)	Y–Te3 ^vi^	3.052(1)	Cu–Te3 ^i^	2.673(1)
Eu–Te2 ^iv^	3.3552(6)	Y–Te1 ^vii^	3.0661(9)		
Eu–Te2^i ii^	3.3552(6)	Y–Te1 ^viii^	3.0661(9)		
Eu–Te1 ^v^	3.6442(8)				
**Bond angles**					
Te3^i^–Eu–Te3 ^ii^	81.58(2)	Te3–Y–Te2 ^iv^	92.74(3)	Te2–Cu–Te1	104.24(5)
Te3^i^–Eu–Te1 ^iii^	144.84(2)	Te3–Y–Te2 ^iii^	92.74(3)	Te2–Cu–Te3 ^ii^	112.46(3)
Te3 ^ii^–Eu–Te1 ^iii^	88.46(1)	Te2 ^iv^–Y–Te2 ^iii^	90.18(3)	Te1–Cu–Te3 ^ii^	109.89(3)
Te3 ^i^–Eu–Te1 ^iv^	88.46(1)	Te3–Y–Te3 ^vi^	178.03(4)	Te2–Cu–Te3 ^i^	112.46(3)
Te3 ^ii^–Eu–Te1 ^iv^	144.84(2)	Te2 ^iv^–Y–Te3 ^vi^	88.66(3)	Te1–Cu–Te3 ^i^	109.89(3)
Te1 ^iii^–Eu–Te1 ^iv^	80.53(2)	Te2 ^iii^–Y–Te3 ^vi^	88.66(3)	Te3 ^ii^–Cu–Te3 ^i^	107.86(6)
Te3 ^i^–Eu–Te2 ^iv^	81.17(2)	Te3–Y–Te1 ^vii^	87.61(3)		
Te3 ^ii^–Eu–Te2 ^iv^	133.46(2)	Te2 ^iv^–Y–Te1 ^vii^	179.55(3)		
Te1 ^iii^–Eu–Te2 ^iv^	127.57(2)	Te2 ^iii^–Y–Te1 ^vii^	90.10(1)		
Te1 ^iv^–Eu–Te2 ^iv^	77.14(2)	Te3 ^vi^–Y–Te1 ^vii^	90.99(2)		
Te3 ^i^–Eu–Te2 ^iii^	133.46(2)	Te3–Y–Te1 ^viii^	87.61(3)		
Te3 ^ii^–Eu–Te2 ^iii^	81.17(2)	Te2 ^iv^–Y–Te1 ^viii^	90.10(1)		
Te1 ^iii^–Eu–Te2 ^iii^	77.14(2)	Te2 ^iii^–Y–Te1 ^viii^	179.55(3)		
Te1 ^iv^–Eu–Te2 ^iii^	127.57(2)	Te3 ^vi^–Y–Te1 ^viii^	90.99(2)		
Te2 ^iv^–Eu–Te2 ^iii^	80.18(2)	Te1 ^vii^–Y–Te1 ^viii^	89.61(3)		
Te3 ^i^–Eu–Te1 ^v^	74.77(2)				
Te3 ^ii^–Eu–Te1 ^v^	74.77(2)				
Te1 ^iii^–Eu–Te1 ^v^	70.07(2)				
Te1 ^iv^–Eu–Te1 ^v^	70.07(2)				
Te2 ^iv^–Eu–Te1 ^v^	139.38(1)				
Te2 ^iii^–Eu–Te1 ^v^	139.38(1)				
Symmetry codes: (i) −*x* + ^1^/_2_, −*y*, *z*−^1^/_2_; (ii) −*x* + ^1^/_2_, −*y* + 1, *z*−^1^/_2_; (iii) −*x* + ^1^/_2_, −*y* + 1, *z* + ^1^/_2_; (iv) −*x* + ^1^/_2_, −*y*, *z* + ^1^/_2_; (v) *x* + ^1^/_2_, *y*, −*z* + ^1^/_2_; (vi) *x*−^1^/_2_, *y*, −*z* + ^3^/_2_; (vii) −*x*, −*y* + 1, −*z* + 1; (viii) −*x*, −*y*, −*z* + 1					
***β*** **-EuYCuTe_3_**					
**Bond lengths**					
Eu–Te1 ^i^	3.296(1)	Y–Te1 ^v^	3.0501(6)	Cu–Te2 ^x^	2.645(2)
Eu–Te1 ^ii^	3.296(1)	Y–Te1	3.0501(6)	Cu–Te2	2.645(2)
Eu–Te2 ^iii^	3.350(1)	Y–Te2 ^vi^	3.0609(8)	Cu–Te1 ^ii^	2.676(2)
Eu–Te2 ^i^	3.350(1)	Y–Te2 ^vii^	3.0609(8)	Cu–Te1 ^i^	2.676(2)
Eu–Te2 ^ii^	3.350(1)	Y–Te2 ^viii^	3.0609(7)		
Eu–Te2 ^iv^	3.350(1)	Y–Te2 ^ix^	3.0609(7)		
**Bond angles**					
Te1 ^i^–Eu–Te1 ^ii^	81.96(4)	Te1 ^v^–Y–Te2 ^vi^	91.84(3)	Te2 ^x^–Cu–Te2	104.3(1)
Te1 ^i^–Eu–Te2 ^iii^	139.14(2)	Te1–Y–Te2 ^vi^	88.16(3)	Te2 ^x^–Cu–Te1 ^ii^	111.19(2)
Te1 ^ii^–Eu–Te2 ^iii^	84.84(2)	Te1 ^v^–Y–Te2 ^vii^	88.16(3)	Te2–Cu–Te1 ^ii^	111.19(2)
Te1 ^i^–Eu–Te2 ^i^	84.84(2)	Te1–Y–Te2 ^vii^	91.84(3)	Te2 ^x^–Cu–Te1 ^i^	111.19(2)
Te1 ^ii^–Eu–Te2^i^	139.14(2)	Te2 ^vi^–Y–Te2 ^vii^	180.00(5)	Te2–Cu–Te1 ^i^	111.19(2)
Te2 ^iii^–Eu–Te2^i^	127.62(5)	Te1 ^v^–Y–Te2 ^viii^	88.16(3)	Te1 ^ii^–Cu–Te1 ^i^	107.8(1)
Te1 ^i^–Eu–Te2 ^ii^	139.14(2)	Te1–Y–Te2 ^viii^	91.84(3)		
Te1 ^ii^–Eu–Te2 ^ii^	84.84(2)	Te2 ^vi^–Y–Te2 ^viii^	90.15(3)		
Te2 ^iii^–Eu–Te2 ^ii^	77.16(3)	Te2 ^vii^–Y–Te2 ^viii^	89.85(3)		
Te2 ^i^–Eu–Te2 ^ii^	80.37(3)	Te1 ^v^–Y–Te2 ^ix^	91.84(3)		
Te1 ^i^–Eu–Te2 ^iv^	84.84(2)	Te1–Y–Te2 ^ix^	88.16(3)		
Te1 ^ii^–Eu–Te2 ^iv^	139.14(2)	Te2 ^vi^–Y–Te2 ^ix^	89.85(3)		
Te2 ^iii^–Eu–Te2 ^iv^	80.37(3)	Te2 ^vii^–Y–Te2 ^ix^	90.15(3)		
Te2 ^i^–Eu–Te2 ^iv^	77.16(3)	Te2 ^viii^–Y–Te2 ^ix^	180.00(3)		
Te2 ^ii^–Eu–Te2 ^iv^	127.62(5)	Te1 ^v^–Y–Te1	180.0		
Symmetry codes: (i) *x*−^1^/_2_, *y* + ^1^/_2_, *z*; (ii) *x* + ^1^/_2_, *y* + ^1^/_2_, *z*; (iii) *x* + ^1^/_2_, *y* + ^1^/_2_, −*z* + ^1^/_2_; (iv) *x*−^1^/_2_, *y* + ^1^/_2_, −*z* + ^1^/_2_; (v) −*x*, −*y*, −*z*; (vi) −*x* + ^1^/_2_, −*y* + ^1^/_2_, −*z*; (vii) *x*−^1^/_2_, *y*−^1^/_2_, *z*; (viii) *x* + ^1^/_2_, *y*−^1^/_2_, *z*; (ix) −*x*−^1^/_2_, −*y* + ^1^/_2_, −*z*; (x) *x*, *y*, −*z* + ^1^/_2_					

**Table 3:** Elastic constants, bulk modulus (*B*), shear modulus (*G*), *Young’s* modulus (*Y*), *Poisson’s* ratio (P.r.) and universal anisotropy index (*A*^U^) of EuYCuTe_3_.

Space Group	C_11_	C_12_	C_13_	C_22_	C_23_	C_33_	C_44_	C_55_	C_66_	Averaging Scheme	*B*	*Y*	*G*	P.r.	*A* ^U^
	116	52	38	90	43	110	5	30	40	*Voigt*	64.1	71.9	27.4	0.313	4.02
*Cmcm*										*Reuss*	63.7	42.2	15.2	0.390	
										*Hill*	63.9	57.5	21.3	0.350	
	100	42	43	113	47	88	36	15	29	*Voigt*	62.8	71.5	27.3	0.310	
*Pnma*										*Reuss*	62.2	65.4	24.7	0.325	0.54
										*Hill*	62.5	68.5	26.0	0.318	

**Table 4:** Fractional atomic coordinates in a single-layer EuYCuTe_3_ structure in relative units for the *x*/*a* and *y*/*b* coordinates and in Å for the off-plane *z* coordinate. Optimized lattice parameters are *a* = 4.386 Å, *b* = 11.832 Å.

Atom	Site	*x*/*a*	*y*/*b*	*z*, Å
Eu	2*b*	^1^/_2_	0.235	2.443
Y	2*b*	^1^/_2_	0.483	0.038
Cu	2*a*	0	0.1711	0.067
Te1	2*b*	^1^/_2_	0.7369	0.941
Te2	2*a*	0	0.4323	2.072
Te3	2*a*	0	0.03159	2.077

## Data Availability

The original contributions presented in this study are included in the article/supplementary material. Further inquiries can be directed to the corresponding author.

## Supplementary Materials

The following supporting information can be downloaded at: https://www.mdpi.com/article/10.3390/ma18040820/s1. Table S1. Fractional atomic coordinates and equivalent isotropic displacement parameters of *α*- and *β*-EuYCuTe_3._ Table S2. Anisotropic displacement parameters (Å^2^) of *α*- and *β*-EuYCuTe_3_. Table S3. Wavenumbers and types of phonon modes at the Γ-point for EuYCuTe_3_ (*Cmcm* form). The intensity of the Raman modes was calculated for *λ* = 532 nm and *T* = 300 K. Table S4. Wavenumbers (cm^−1^) and types of the phonon modes at the Γ-point for EuYCuTe_3_ (*Pnma* form). The intensity of the Raman modes was calculated for *λ* = 532 nm and *T* = 300 K. Figure S1. The calculated in DFT+U approximation band structure (left) and projected on atomic species density of states (right) for the *Cmcm*-phase of EuYCuTe_3_. Figure S2. The calculated heat capacity (blue curve) at constant volume and *Debye* model *C*_v_ with fitted *T*_D_ value. The *Dulong*-*Petit* limit of 3R is plotted as gray dashed line.

## References

[1] Fiori G., Bonaccorso F., Iannaccone G., Palacios T., Neumaier D., Seabaugh A., Banerjee S.K., Colombo L. (2014). Electronics based on two-dimensional materials. Nat. Nanotechnol..

[2] Navarro-Moratalla E., Jarillo-Herrero P. (2016). Two-dimensional superconductivity: The Ising on the monolayer. Nat. Phys..

[3] Cui S., Pu H., Wells S.A., Wen Z., Mao S., Chang J., Hersam M.C., Chen J. (2015). Ultrahigh sensitivity and layer-dependent sensing performance of phosphorene-based gas sensors. Nat. Commun..

[4] Maier S., Prakash J., Berthebaud D., Perez O., Bobev S., Gascoin F. (2016). Crystal structures of the four new quaternary copper(I)-selenides A_0.5_CuZrSe_3_ and ACuYSe_3_ (A = Sr, Ba). J. Solid State Chem..

[5] Pal K., Xia Y., Shen J., He J., Luo Y., Kanatzidis M.G., Wolverton C. (2021). Accelerated discovery of a large family of quaternary chalcogenides with very low lattice thermal conductivity. Comput. Mater..

[6] Pal K., Hua X., Xia Y., Wolverton C. (2019). Unraveling the structure-valence-property relationships in AMM′Q_3_ chalcogenides with promising thermoelectric performance. ACS Appl. Energy Mater..

[7] Yang Y., Ibers J.A. (1999). Synthesis and characterization of a series of quaternary chalcogenides BaLnMQ_3_ (Ln = rare earth, M = coinage metal, Q = Se or Te). J. Solid State Chem..

[8] Wakeshima M., Furuuchi F., Hinatsu Y. (2004). Crystal structures and magnetic properties of novel rare-earth copper sulfides, EuRCuS_3_ (R = Y, Gd–Lu). J. Phys. Condens. Matter..

[9] Gulay L.D., Shemet V.Y., Olekseynuk I.D. (2004). Crystal structures of the compounds YCuPbSe_3_, Y_3_CuSnSe_7_ and Y_3_Cu_0.685_Se_6_. J. Alloys Compd..

[10] Babo J.-M., Schleid T. (2010). CsCu_2_Sc_3_Te_6_ and CsCuY_2_Te_4_: Two new quaternary cesium copper rare-earth metal tellurides. Solid State Sci..

[11] Ruseikina A.V., Andreev O.V. (2017). Regularities of Change in the Structural Parameters of EuLnCuS_3_ (Ln = La–Nd, Sm, Gd, Ho). Russ. J. Inorg. Chem..

[12] Ruseikina A.V., Solovyev L.A., Andreev O.V., Kislitsyn A.A. (2014). EuNdCuS_3_: Crystal Structure of the High-Temperature Polymorph and Properties. Russ. J. Inorg. Chem..

[13] Ruseikina A.V., Demchuk Z.A. (2017). Crystal Structure and Properties of AHoCuS_3_ (A = Sr or Eu). Russ. J. Inorg. Chem..

[14] Christuk A.E., Wu P., Ibers J.A. (1994). New Quaternary Chalcogenides BaLnMQ_3_ (Ln = Rare Earth; M = Cu, Ag; Q = S, Se). Structures and Grinding-lnduced Journal Pre-proof 34 Phase Transition in BaLaCuQ_3_. J. Solid State Chem..

[15] Yao J.-Y., Deng B., Ellis D.E., Ibers J.A. (2003). Syntheses, structures, physical properties, and electronic structures of KLn_2_CuS_4_ (Ln = Y, Nd, Sm, Tb, Ho) and K_2_Ln_4_Cu_4_S_9_ (Ln = Dy, Ho). J. Solid State Chem..

[16] Babo J.-M. (2010). Syntheses and Crystal Structures of Quaternary Chalcogenides Containing Rare Earth and Coinage Metals. Ph.D. Thesis.

[17] Yadav S., Prakash J. (2024). Synthesis and crystal structure of Ba_2_Y_0.87(1)_Mn_1.71(1)_Te_5_. Acta Crystallogr..

[18] Yao J., Deng B., Sherry L.J., McFarland A.D., Ellis D.E., van Duyne R.P., Ibers J.A. (2004). Syntheses, Structure, Some Band Gaps, and Electronic Structures of CsLnZnTe_3_ (Ln = La, Pr, Nd, Sm, Gd, Tb, Dy, Ho, Er, Tm, Y). Inorg. Chem..

[19] Yin W., Wang W., Bai L., Feng K., Shi Y., Hao W., Yao J., Wu Y. (2012). Syntheses, Structures, Physical Properties, and Electronic Structures of Ba_2_MLnTe_5_ (M = Ga and Ln = Sm, Gd, Dy, Er, Y.; M = In and Ln = Ce, Nd, Sm, Gd, Dy, Er, Y). Inorg. Chem..

[20] Mitchell K., Huang F.Q., McFarland A.D., Haynes C.L., Somers R.C., Van Duyne R.P., Ibers J.A. (2003). The CsLnMSe_3_ Semiconductors (Ln = Rare-Earth Element, Y.; M = Zn, Cd, Hg). Inorg. Chem..

[21] Mitchell K., Haynes C.L., McFarland A.D., Van Duyne R.P., Ibers J.A. (2002). Tuning of Optical Band Gaps: Syntheses, Structures, Magnetic Properties, and Optical Properties of CsLnZnSe_3_ (Ln = Sm, Tb, Dy, Ho, Er, Tm, Yb, and Y). Inorg. Chem..

[22] Shannon R.D. (1976). Revised effective ionic radii and systematic studies of interatomic distances in halides and chalcogenides. Acta Crystallogr..

[23] Azarapin N.O. (2022). Synthesis, Structure and Properties of Compounds BaRECuS_3_ (RE = Rare Earth Element). Ph.D. Thesis.

[24] Ibers J.A., Christuk A.E., Wu P. (1994). New quaternary chalcogenides BaLnMQ_3_ (Ln = rare earth or Sc; M = Cu, Ag; Q = S, Se). II. Structure and property variation vs rare-earth element. J. Solid State Chem..

[25] Eberle M.A., Schleid T. (2016). Expanding the SrCuRES_3_ Series with the Rare-Earth Metals Scandium and Yttrium. Z. Kristallogr..

[26] Ruseikina A.V., Grigoriev M.V., Solovyov L.A., Molokeev M.S., Garmonov A.A., Velikanov D.A., Safin D.A. (2023). Unravelling the rear-earth (RE) element-induced magnetic and optical properties in the structures of quaternary selenides SrRECuSe_3_. Inorg. Chem. Comm..

[27] Grigoriev M.V., Solovyov L.A., Ruseikina A.V., Aleksandrovsky A.S., Chernyshev V.A., Velikanov D.A., Garmonov A.A., Molokeev M.S., Oreshonkov A.S., Shestakov N.P. (2022). Quaternary Selenides EuLnCuSe_3_: Synthesis, Structures, Properties and In Silico Studies. Int. J. Mol. Sci..

[28] Ishtiyak M., Jana S., Karthikeyan R., Ramesh M., Tripathy B., Malladi S.K., Niranjan M.K., Prakash J. (2021). Syntheses of five new layered quaternary chalcogenides SrScCuSe_3_, SrScCuTe_3_, BaScCuSe_3_, BaScCuTe_3_, and BaScAgTe_3_: Crystal structures, thermoelectric properties, and electronic structures. Inorg. Chem. Front..

[29] Eickmeier K., Poschkamp R., Dronskowski R., Steinberg S. (2022). Exploring the impact of lone pairs on the structural features of alkaline-earth (A) transition-metal (M,M’) chalcogenides (Q) AMM’Q_3_. Eur. J. Inorg. Chem..

[30] Benaadad M., Nafidi A., Melkoud S., Khan M.S., Soubane D. (2021). First-principles investigations of structural, optoelectronic and thermoelectric properties of Cu-based chalcogenides compounds. J. Mater. Sci..

[31] Sheldrick G.M. (2008). A short history of SHELX. Acta Crystallogr..

[32] Spek A.L. (2008). PLATON—A Multipurpose Crystallographic Tool.

[33] Dovesi R., Saunders V.R., Roetti C., Orlando R., Zicovich-Wilson C.M., Pascale F., Civalleri B., Doll K., Harrison N.M., Bush I.J. 2018 CRYSTAL17 User’s Manual. http://www.crystal.unito.it/Manuals/crystal17.pdf.

[34] Crystal. http://www.crystal.unito.it/index.php.

[35] Gonze X., Beuken J.M., Caracas R., Detraux F., Fuchs M., Rignanese G.M., Sindic L., Verstraete M., Zerah G., Jollet F. (2002). First-principles computation of material properties: The ABINIT software project. Comput. Mater. Sci..

[36] Dolg M., Stoll H., Savin A., Preuss H. (1989). Energy-adjusted pseudopotentials for the rare earth elements. Theor. Chim. Acta.

[37] Dolg M., Stoll H., Preuss H.A. (1993). Combination of quasi-relativistic pseudo-potential and ligand field calculations for lanthanoid compounds. Theor. Chim. Acta.

[38] Energy-consistent Pseudopotentials of the Stuttgart/Cologne Group. http://www.tc.uni-koeln.de/PP/clickpse.en.html.

[39] Towler M. CRYSTAL Resources Page. https://vallico.net/mike_towler/crystal.html.

[40] Dovesi R., Erba A., Orlando R., Zicovich-Wilson C.M., Civalleri B., Maschio L., Rérat M., Casassa S., Baima J., Salustro S. (2018). Quantum-mechanical condensed matter simulations with CRYSTAL. Wiley Interdiscipl. Rev. Comput. Mol. Sci..

[41] Becke A.D. (1993). Density-Functional Thermochemistry. III. The Role of Exact Exchange. J. Chem. Phys..

[42] De la Pierre M., Orlando R., Maschio L., Doll K., Ugliengo P., Dovesi R. (2011). Performance of six functionals (LDA, PBE, PBESOL, B3LYP, PBE0, and WC1LYP) in the simulation of vibrational and dielectric properties of crystalline compounds. The case of forsterite Mg_2_SiO_4_. J. Comput. Chem..

[43] Togo A., Chaput L., Tadano T., Tanaka I. (2023). Implementation strategies in phonopy and phono3py. J. Phys. Condens. Matter.

[44] Hjorth Larsen A., Jørgen Mortensen J., Blomqvist J., Castelli I.E., Christensen R., Dułak M., Friis J., Groves M.N., Hammer B., Hargus C. (2017). The atomic simulation environment—A Python library for working with atoms. J. Phys. Condens. Matter.

[45] Yang L., Powell D.R., Houser R.P. (2007). Structural variation in copper(I) complexes with pyridylmethylamide ligands: Structural analysis with a new four-coordinate geometry index, τ_4_. Dalton Trans..

[46] Ruseikina A.V., Solov’ev L.A., Andreev O.V. (2013). Crystal Structures of *α-* and *β-*EuPrCuS_3_. Russ. J. Inorg. Chem..

[47] Ruseikina A.V., Solov’ev L.A. (2016). Crystal Structures of *α*- and *β*-SrCeCuS_3_. Russ. J. Inorg. Chem..

[48] Hinuma Y., Pizzi G., Kumagai Y., Oba F., Tanaka I. (2017). Band structure diagram paths based on crystallography. Comput. Mater. Sci..

[49] Aroyo M.I., Kirov A., Capillas C., Perez-Mato J.M., Wondratschek H. (2006). Bilbao Crystallographic Server II: Representations of crystallographic point groups and space groups. Acta Crystallogr..

[50] Morelli D.T., Slack G.A., Shindé S.L., Goela J.S. (2006). High Lattice Thermal Conductivity Solids. High Thermal Conductivity Materials.

[51] Ranganathan S.I., Ostoja-Starzewski M. (2008). Universal Anisotropy Index. Phys. Rev. Lett..

[52] De la Flor G., Orobengoa D., Evarestov R.A., Kitaev Y., Tasci E., Aroyo M.I. (2019). The site-symmetry induced representations of layer groups on the Bilbao Crystallographic Server. J. Appl. Crystallogr..

[53] Choudhary K., Kalish I., Beams R., Tavazza F. (2017). High-throughput Identification and Characterization of Two-dimensional Materials using Density functional theory. Sci. Rep..

[54] Sansone G., Maschio L., Usvyat D., Schütz M., Karttunen A. (2016). Toward an Accurate Estimate of the Exfoliation Energy of Black Phosphorus: A Periodic Quantum Chemical Approach. J. Phys. Chem. Lett..

[55] Benedict L.X., Chopra N.G., Cohen M.L., Zettl A., Louie S.G., Crespi V.H. (1998). Microscopic Determination of the Interlayer Binding Energy in Graphite. Chem. Phys. Lett..

