# Supplementary material for: Theoretical and Experimental Studies of the Structural Chameleon EuYCuTe3
Source: Materials (Basel). 2025 Feb 13;18(4):820. doi: 10.3390/ma18040820 (PMC11857737; doi:10.3390/ma18040820)
Supplement: Supplementary file 1 [file materials-18-00820-s001.zip › materials-3427858-supplementary.pdf]

Supplementary Materials

Theoretical and Experimental Studies of the Structural Chameleon $EuYCuTe_3$

Anna V. Ruseikina [1,*], Maxim V. Grigoriev [1,2], Vladimir A. Chernyshev [3], Evgenii M. Roginskii [4], Alexander A. Garmonov [1], Ralf J. C. Locke [2] and Thomas Schleid [2,*]

[1] School of Natural Sciences, University of Tyumen, 625003 Tyumen, Russia
[2] Institute for Inorganic Chemistry, University of Stuttgart, D-70569 Stuttgart, Germany
[3] Institute of Natural Sciences and Mathematics, Ural Federal University Named after the First President of Russia B.N. Yeltsin, Ekaterinburg 620002, Russia
[4] Ioffe Institute, Politekhnicheskaya 26, St. Petersburg 194021, Russia
* Correspondence: a.v.rusejkina@utmn.ru (A.V.R.); thomas.schleid@iac.uni-stuttgart.de (T.S.)

Table S1. Fractional atomic coordinates and equivalent isotropic displacement parameters of α- and β-$EuYCuTe_3$.

Atom	*x* / *a*	*y* / *b*	*z* / *c*	$U_{eq}{}^*$ (Å^2)
		**α-$EuYCuTe_3$**		
Eu	0.26498(5)	1/4	0.50455(4)	0.02269(18)
Y	0.01270(8)	1/4	0.74670(7)	0.0183(2)
Cu	0.24248(12)	1/4	0.22111(11)	0.0271(3)
Te1	0.05323(6)	1/4	0.11060(5)	0.01762(19)
Te2	0.42360(6)	1/4	0.10496(5)	0.01842(19)
Te3	0.26063(6)	1/4	0.83093(5)	0.01738(19)
		**β-$EuYCuTe_3$**		
Eu	0	0.75451(8)	1/4	0.0398(4)
Y	0	0	0	0.0316(6)
Cu	0	0.47087(19)	1/4	0.0352(7)
Te1	0	0.08090(9)	1/4	0.0283(4)
Te2	0	0.35768(7)	0.06486(8)	0.0308(4)

$^*U_{eq} = 1/3(U_{11} + U_{22} + U_{33})$ for both.

Table S2. Anisotropic displacement parameters (Å^2) of α- and β-$EuYCuTe_3$.

	U_{11}	U_{22}	U_{33}	U_{12}	U_{13}	U_{23}
			**α-$EuYCuTe_3$**			
Eu	0.0303(3)	0.0155(3)	0.0223(3)	0	0.0004(2)	0
Y	0.0194(5)	0.0162(5)	0.0192(6)	0	0.0001(4)	0
Cu	0.0288(7)	0.0261(7)	0.0266(8)	0	−0.0007(5)	0
Te1	0.0202(3)	0.0154(3)	0.0173(4)	0	−0.0001(2)	0
Te2	0.0220(3)	0.0153(3)	0.0179(4)	0	−0.0011(3)	0
Te3	0.0172(3)	0.0164(3)	0.0185(4)	0	0.0002(2)	0
			**β-$EuYCuTe_3$**			
Eu	0.0246(6)	0.0304(7)	0.0644(9)	0	0	0
Y	0.0260(10)	0.0278(12)	0.0411(13)	0	0	−0.0081(9)
Cu	0.0362(14)	0.0335(16)	0.0358(15)	0	0	0
Te1	0.0269(7)	0.0262(8)	0.0319(8)	0	0	0
Te2	0.0252(6)	0.0263(6)	0.0409(7)	0	0	−0.0053(4)

Table S3. Wavenumbers and types of phonon modes at the Γ-point for $EuYCuTe_3$ (*Cmcm* form). The intensity of the Raman modes was calculated for λ = 532 nm and T = 300 K.

**Wavenumber (cm^{-1})**	**Irreps**	**IR modes**		**Raman modes**		**Involved ions**
		**Active / Inactive**	**Intensity ($km \cdot mol^{-1}$)**	**Active / Inactive**	**Intensity (rel. un.)**	
32	B_{1u}	A	38	I		Eu, Y^S, Cu^S, Te1, $Te2^S$
38	B_{1u}	A	9	I		Eu^S, Y^S, Cu^W, $Te1^S$, Te2
46	A_u	I	0	I		Y^S, $Te2^S$
53.8	B_{2u}	A	20	I		Y^S, Cu^S, $Te1^S$, Te2
54.4	B_{1g}	I	0	A	736	Eu^S, Cu^S, $Te1^S$, $Te2^W$
57	A_g	I	0	A	712	Eu^S, Cu^S, $Te1^S$, Te2
58	B_{2g}	I	0	A	396	Eu^S, Cu, Te2
71	B_{2g}	I	0	A	336	Eu, Cu^S, Te2
81	B_{3u}	A	129	I		Eu^S, Y^W, Cu^S, $Te1^W$, Te2
82	A_g	I	0	A	293	Eu^S, Cu^S, $Te1^W$, Te2
83	B_{1g}	I	0	A	168	Eu^S, Cu^S, Te1, $Te2^W$
87	B_{1u}	A	79	I		Eu^S, Y, Cu, Te2
89	B_{2u}	A	9	I		Eu^S, Y, Cu^S
91	B_{3u}	A	67	I		Eu, Y, Cu, Te1, Te2
109	B_{3g}	I	0	A	215	$Te2^S$
115	B_{1g}	I	0	A	25	Eu, Cu^W, $Te2^S$
116	B_{3u}	A	68	I		Eu, Y, Cu^S, $Te1^S$, $Te2^W$
117	B_{2g}	I	0	A	10	Eu, Cu, Te1, Te2
120	B_{1u}	A	302	I		Y, Cu^S, Te2
125	B_{3u}	A	19	I		Eu^W, Y, Cu^S, $Te1^W$, Te2
129	A_g	I	0	A	109	Eu, Te1, Te2
131	A_u	I	0	I		Y^S, Te2
132	B_{2u}	A	36	I		Y, Cu^S, $Te1^S$, $Te2^W$
134.6	B_{2u}	A	781	I		Eu^W, Y^S, Cu, Te1, Te2
135.5	B_{1g}	I	0	A	63	Eu^W, Cu^S, $Te1^S$
139	B_{2g}	I	0	A	211	Eu^W, Cu^S, Te1, Te2
140	A_g	I	0	A	70	Cu^S, Te1, Te2
148	A_g	I	0	A	1000	Cu, Te1, Te2
152	B_{1u}	A	334	I		Y^S, Cu^W, Te1,
154	B_{2g}	I	0	A	38	Cu, Te1, Te2
170	B_{1u}	A	31	I		Y^S, Cu, $Te2^W$
177	B_{3u}	A	154	I		Y^S, Cu, $Te1^W$, $Te2^W$
183	B_{3u}	A	83	I		Y^S, $Te2^W$

Note. The displacements of ions in phonon modes reach 0.03–0.04 Å. Indexes "S" and "W" at last column are «strong» or «weak» displacements of ion in the mode, accordingly. The displacement noted as "S" if it value greater than or equal to 0.02 Å. The displacement noted as "W" if it value does not exceed 0.01 Å. The ion is does not mentioned in the column "Involved ions" if the displacement is less than 0.005 Å.

Table S4. Wavenumbers (cm^{-1}) and types of the phonon modes at the Γ-point for $EuYCuTe_3$ (*Pnma* form). The intensity of the Raman modes was calculated for λ = 532 nm and T = 300 K

Wavenumber (cm^{-1})	Irreps	IR modes		Raman modes		Participating ions
		Active / Inactive	Intensity, (km·mol^{-1})	Active / Inactive	Intensity (rel. un.)	
22.2	A_g	I	0	A	476	Y^S, Eu^S, Cu, Te1, $Te2^S$, $Te3^S$
34.8	B_{1g}	I	0	A	52	Y^S, Eu, Cu^S, Te1, Te2, Te3
38.3	B_{2g}	I	0	A	175	Y, Eu^S, Cu, Te1, Te2, Te3
41.0	B_{3u}	A	42.83	I		Y, Eu, Cu, Te1, $Te2^S$, Te3
43.5	A_g	I	0	A	229	Y, Eu, Cu, Te1, Te2, Te3
45.5	B_{1g}	I	0	A	175	Y, Cu^S, Te1, Te2, $Te3^S$
45.9	A_u	I	0	I		Y^S, Eu^W, Cu, $Te1^S$, Te2, $Te3^W$
49.9	B_{3g}	I	0	A	227	Y^S, Eu, Cu, Te1, Te2, Te3
51.3	B_{3u}	A	24.92	I		Y, Eu^W, Cu^S, Te1, $Te2^W$, Te3
51.5	B_{2u}	A	34.28	I		Y, Cu^S, Te1, $Te2^W$, Te3
52.7	B_{1u}	A	0.1	I		Eu^S, Cu, Te1, Te2
53.5	B_{3g}	I	0	A	1000	Y, Eu, Cu^S, Te1, $Te2^W$, Te3
54.8	A_u	I	0	I		Y^W, Eu, Cu^S, Te2, Te3
56.4	A_g	I	0	A	653	Eu, Cu^S, Te1, $Te2^W$, Te3
61.4	B_{3u}	A	0.02	I		Y, Eu, Cu, Te1, Te2, Te3
63.2	A_g	I	0	A	659	Y, Eu, Cu^S, $Te1^W$, Te2, Te3
64.3	B_{2g}	I	0	A	576	Y^W, Eu^S, Cu, $Te1^W$, Te2
70.1	B_{1u}	A	11.56	I		Eu, Cu^S, Te1, Te2
71.8	B_{2g}	I	0	A	34	Y, Eu, Cu, $Te1^W$, Te2, Te3
74.1	A_u	I	0	I		Eu, Cu^S, Te1, $Te2^W$, $Te3^W$
76.3	B_{2g}	I	0	A	516	Cu^S, Te1, Te2
78.4	A_g	I	0	A	360	Y^W, Eu, Cu, $Te1^W$, Te2, $Te3^W$
79.7	B_{3g}	I	0	A	132	Eu, Cu^S, $Te1^W$, $Te2^W$, $Te3^W$
83.4	B_{1u}	A	197.5	I		Y^W, Eu, Cu^S, Te1, $Te2^W$, $Te3^W$
86.0	B_{1g}	I	0	A	27	Y, Eu^S, Cu
87.7	B_{2u}	A	17.82	I		Y, Eu^S, Cu, $Te2^W$
89.5	B_{3u}	A	142.21	I		Y^W, Eu, Cu, $Te1^W$, Te2
90.6	B_{3u}	A	14.84	I		Eu, Cu, $Te1^W$, $Te2^W$
91.7	B_{1u}	A	213.79	I		Y, Eu, Cu, Te1, Te2, $Te3^W$
96.5	B_{2g}	I	0	A	57	Y^W, Eu, Cu, $Te1^W$, Te2
96.7	A_g	I	0	A	39	Y^W, Eu, Cu^W, Te1, Te2
105.3	B_{2u}	A	6.8	I		Te1, Te2
106.1	B_{1u}	A	47.7	I		Y, Eu^W, Cu^W, $Te1^W$, Te2, Te3
107.4	B_{1g}	I	0	A	304	Te1, Te2
113.2	B_{2g}	I	0	A	3.16	Y^W, Eu^W, Cu^W, Te1, $Te2^W$, Te3
113.5	A_u	I	0	I		Eu^W, Cu^W, Te1, Te2,
114.1	B_{3g}	I	0	A	35	Eu^W, Te1, Te2
118.9	B_{1u}	A	45.93	I		Y^W, Cu, $Te1^W$, $Te2^W$, Te3
119.0	B_{3u}	A	548.9	I		Y^W, Cu^S, Te1, $Te2^W$, $Te3^W$
119.2	A_g	I	0	A	80	Y^W, Cu^S, $Te1^W$, $Te2^W$, $Te3^W$
121.5	B_{1u}	A	59.06	I		Y^W, Eu^W, Cu^W, Te1, $Te2^W$, Te3
121.8	B_{2g}	I	0	A	50	Y^W, Eu^W, Cu^W, $Te1^W$, $Te2^W$, Te3
124.2	B_{2g}	I	0	A	6.10	Y^W, Cu, Te1, $Te2^W$, $Te3^W$

131.0	A_g	I	0	A	196	Eu^W, Cu^W, Te1, $Te2^W$, $Te3^W$
132.1	A_u	I	0	I		Y^S, $Te1^W$, Te2
132.4	B_{3g}	I	0	A	0.15	Y^S, $Te1^W$, Te2
133.8	B_{1g}	I	0	A	101	Y, Cu, $Te1^W$, $Te2^W$, $Te3^W$
134.1	B_{2u}	A	796.76	I		Y, Cu^W, $Te1^W$, $Te2^W$, $Te3^W$
135.9	B_{3u}	A	5.59	I		Cu^W, Te1, $Te2^W$, Te3
136.5	B_{2u}	A	798.25	I		Y^W, Cu, Te3
137.7	B_{1g}	I	0	A	0.16	Y^W, Eu^W, Cu, $Te1^W$, Te3
138.3	B_{3g}	I	0	A	57	Cu, Te3
138.8	A_u	I	0	I		Cu, Te3
143.7	B_{1u}	A	18.42	I		Y, Cu, $Te1^W$, $Te2^W$, $Te3^W$
144.3	A_g	I	0	A	350	Cu, $Te1^W$, Te2, Te3
147.1	B_{3u}	A	74.11	I		Y, Cu^S, $Te2^W$, $Te3^W$
148.9	B_{3u}	A	120.11	I		Y, Eu^W, Cu^W, $Te1^W$, $Te2^W$, Te3
149.0	B_{2g}	I	0	A	15	Y^W, Cu, $Te1^W$, $Te2^W$, Te3
150.5	A_g	I	0	A	709	Y, Cu, $Te1^W$, $Te2^W$, $Te3^W$
152.7	A_g	I	0	A	482	Y, Cu, $Te1^W$, $Te2^W$, $Te3^W$
152.9	B_{3u}	A	521.6	I		Y, Eu^W, Te1, $Te2^W$, $Te3^W$
156.2	B_{1u}	A	12.82	I		Y^W, Cu, $Te1^W$, $Te2^W$, Te3
158.8	B_{2g}	I	0	A	130	Y^W, Cu, $Te1^W$, Te2, $Te3^W$
172.6	B_{2g}	I	0	A	54	Y^S, Cu^W, $Te3^W$
178.5	B_{3u}	A	24.12	I		Y, Cu, $Te2^W$
179.0	B_{1u}	A	72.17	I		Y, Cu, $Te3^W$
186.2	B_{1u}	A	342.35	I		Y, Cu^W, $Te1^W$, $Te2^W$
188.7	A_g	I	0	A	603	Y, Cu, $Te2^W$
201.8	B_{2g}	I	0	A	1.33	Y, Cu^W, $Te2^W$

Note. The displacements of ions in phonon modes reach 0.03–0.04 Å. Indexes "S" and "W" at last column are strong or weak displacements of ion in the mode, accordingly. The displacement noted as "S" if it value greater than or equal to 0.02 Å. The displacement noted as "W" if it value does not exceed 0.01 Å. The ion is does not mentioned in the column "Involved ions" if the displacement is less than 0.005 Å.

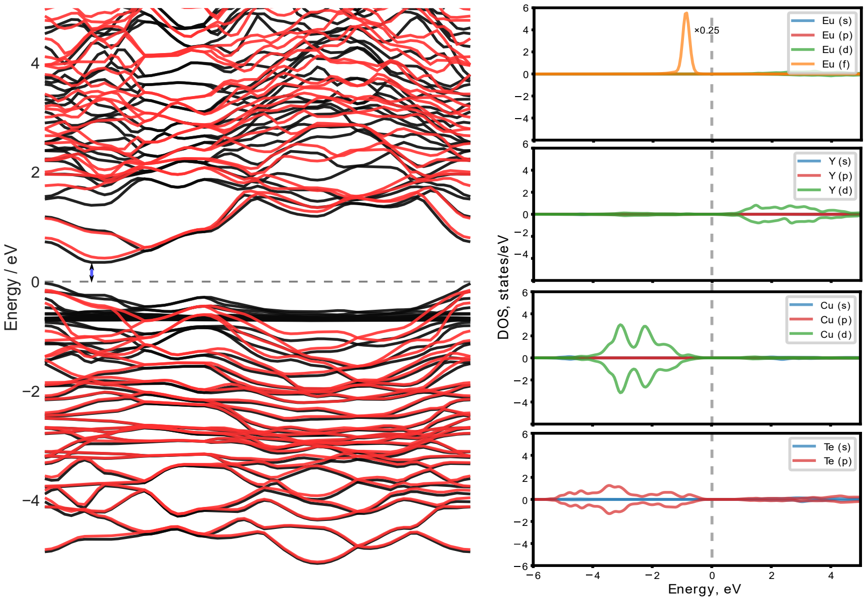

Figure S1. The calculated in DFT+U approximation band structure (left) and projected on atomic species density of states (right) for the *Cmcm*-phase of $EuYCuTe_3$.

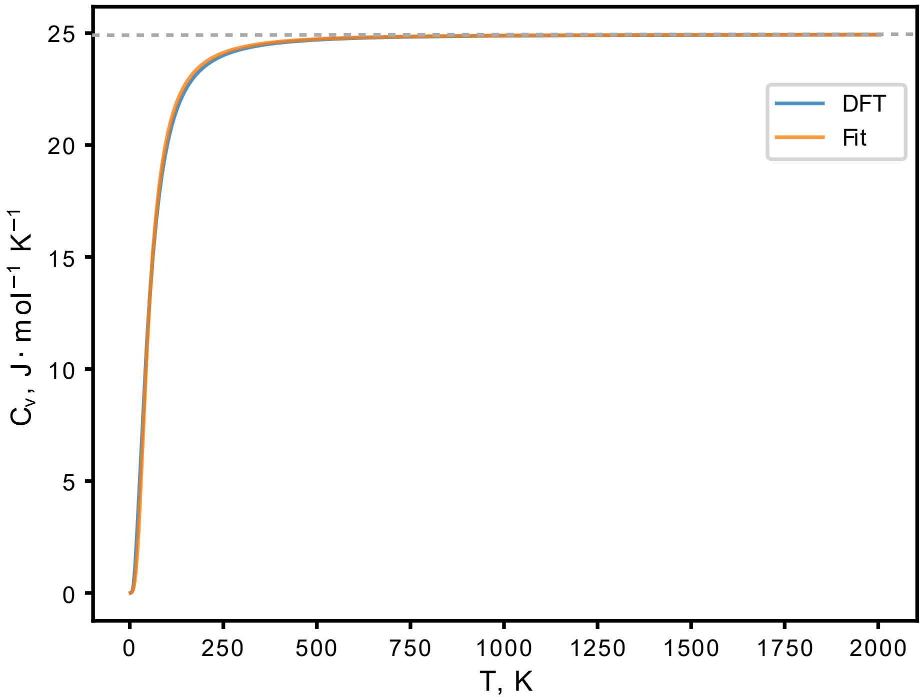

Figure S2. The calculated heat capacity (blue curve) at constant volume and *Debye* model C_v with fitted T_D value. The *Dulong-Petit* limit of 3R is plotted as gray dashed line.
